# Genetic Commonalities Between Metabolic Syndrome and Rheumatic Diseases Through Disease Interactome Modules

**DOI:** 10.1111/jcmm.70329

**Published:** 2025-01-09

**Authors:** Yinli Shi, Shuang Guan, Xi Liu, Hongjun Zhai, Yingying Zhang, Jun Liu, Weibin Yang, Zhong Wang

**Affiliations:** ^1^ Institute of Basic Research in Clinical Medicine China Academy of Chinese Medical Sciences Beijing China; ^2^ Chengdu University of Traditional Chinese Medicine Key Laboratory of Systematic Research of Distinctive Chinese Medicine Resources in Southwest China Chengdu China; ^3^ Institute of Network and Communication Engineering Jinling Institute of Technology Nanjing China; ^4^ Dongzhimen Hospital Beijing University of Chinese Medicine Beijing China; ^5^ Graduate School of China Academy of Chinese Medical Sciences Beijing China

**Keywords:** bioinformatics, disease interactome, disease module, Mendelian randomisation study, metabolic syndrome, rheumatic diseases, translational informatics

## Abstract

This study aims to elucidate the potential genetic commonalities between metabolic syndrome (MetS) and rheumatic diseases through a disease interactome network, according to publicly available large‐scale genome‐wide association studies (GWAS). The analysis included linkage disequilibrium score regression analysis, cross trait meta‐analysis and colocalisation analysis to identify common genetic overlap. Using modular partitioning, the network‐based association between the two disease proteins in the protein–protein interaction set was divided and quantified. Clinical samples from public databases were used to confirm the mapped genes. Mendelian randomisation analyses were conducted using genetic instrumental variables for causal inference. MetS and rheumatoid arthritis (RA), ankylosing spondylitis (AS), systemic lupus erythematosus (SLE), Sjogren's syndrome (SS) and their primary module networks shared topological overlap and genetic correlation. Functional analysis highlighted the significance of these shared targets in processes such as a diverse array of metabolic pathways involving glucose, lipids, energy, protein transport, inflammatory response, autophagy and cytokine regulation, elucidating the pathways through which MetS intersects with rheumatic diseases. Causal associations were determined between MetS phenotypes and rheumatic diseases. The persistence of MetS effects on rheumatic diseases remained evident even after adjusting for alcohol consumption and smoking. We have highlighted specific genetic associations between MetS and rheumatic diseases. Several genes (e.g., PRRC2A, PSMB8, BAG6, GPSM3, PBX2, etc.) have been identified with molecular commonalities in MetS and RA, AS, SLE and SS, which may serve as potential targets for shared treatments.

## 1 Introduction

Rheumatic diseases encompass conditions that cause damage to multiple physiological systems, including bones, joints, endocrine glands, muscles, skin, blood vessels and nerves, often accompanied by immune dysfunction [1]. The classification of these diseases involves categories such as connective tissue, joint‐related, infectious or non‐infectious, metabolic and endocrine, bone‐level, diffuse connective tissue diseases and seronegative spinal arthropathy, with the latter being the most prevalent [2]. The onset and progression of rheumatic diseases are intricately linked to the physiological and pathological mechanisms encompassing immune cell dysfunction, immune tolerance deficiency, inflammatory responses, cell apoptosis, microbiome and intestinal barrier dysfunction and protein glycosylation [3, 4, 5]. A comprehensive transcriptomic and single‐cell RNA‐sequencing study involving patients with rheumatoid arthritis (RA), systemic lupus erythematosus (SLE) and Sjogren's syndrome (SS) revealed shared immune expression characteristics. The dysregulation leading to macrophage expansion and abnormal T cell receptor arrangement has emerged as a potential trigger for the pathogenesis of rheumatic diseases [6]. Abnormal blood pressure, blood glucose and lipid levels may be important risk factors for rheumatic diseases. Obesity, hyperlipidaemia, high blood pressure and impaired glucose tolerance collectively contribute to the aberrant aggregation of metabolic phenotypes, culminating in the development of metabolic syndrome (MetS) [7]. According to the results of a recent Korean study that assessed alcohol consumption over time, variations in alcohol consumption could be associated with an increased risk of developing MetS [8]. Smoking was found to be an independent risk factor for MetS in an additional study. Through the increased production of lipolysis and circulating levels of insulin antagonist hormones (growth hormone, cortisol and catecholamines), smoking may directly impair insulin sensitivity and lead to the development of insulin resistance [9]. Owing to a convergence of numerous risk factors and chronic conditions, MetS has emerged as a prominent and substantial public health concern, exerting a considerable adverse effect on the overall quality of life within the population.

The association between immunity and the neuroendocrine system is robust. Specific chemicals released and their binding with associated receptors can either stimulate or inhibit the activity of immunological and neuroendocrine cells. Metabolic pathways are essential for regulating immune cell function and inflammatory responses, as they support the basic energy needs of cells. In disease states, immune cells undergo metabolic reprogramming, leading to the upregulation of glycolysis, fatty acid oxidation and the pentose phosphate pathway (PPP). Glycolysis provides pyruvate for the tricarboxylic acid (TCA) cycle, which produces adenosine triphosphate (ATP) through oxidative metabolism, while the PPP generates reducing equivalents and biosynthetic precursors [10]. These pathways work in concert to maintain cellular energy homeostasis. Additionally, metabolites such as succinate and reactive oxygen species serve as signalling molecules, directly influencing immune and inflammatory responses [11]. A study has indicated that the onset of RA correlates with reductions in lipid levels, body mass index (BMI), fat and muscle mass, along with alterations in lipid status [12]. In a separate clinical trial, it was observed that individuals with ankylosing spondylitis (AS) exhibited elevated blood pressure and fasting glucose levels, with BMI identified as an independent risk factor for its onset [13]. Patients affected by MetS and obesity typically present with chronic inflammation, characterised by an abundance of immune cells, such as macrophages, T cells, mast cells and cellular inflammation, along with chemokines in their metabolic organs and adipose tissue [14]. The coexistence of chronic inflammation and metabolic alterations represents a risk factor for RA, osteoporosis (OS), osteoarthritis and other rheumatic diseases [15, 16]. More than 100 genes, notably TLR7, IRF7, IL10, AFF1, CSK, IKZF1, SLC15A4, PPP2CA and others, have been linked to the development of autoimmune rheumatic diseases, according to mechanism‐based research. These genes have also been assessed for their significant involvement in metabolic pathways and pathogenesis, including oxidative stress, apoptosis, autophagy, glucose metabolism, lipid metabolism and mitochondrial metabolism [17, 18]. Through immune cells, metabolic genes could impact the immune system and cause rheumatic diseases. However, there remains a gap in the identification and validation of associated genes in this context. Observational studies are vulnerable to confounding factors, necessitating extensive sample sizes and prolonged follow‐up periods, thereby compromising the precision of causal inferences. Consequently, the identification of potential associations between MetS, its phenotypes and rheumatic diseases has become challenging.

The pathogenesis of MetS and numerous rheumatic diseases involves intricate and potent genetic contributions. Various computational methods have recently emerged to help overcome these challenges. Genome‐wide association studies (GWAS), exemplified by genetic correlation analysis, cross‐trait meta‐analysis and Mendelian randomisation (MR), offer comprehensive insights into the overall genetic correlation and shared loci from diverse perspectives [19, 20, 21]. These methodologies serve to validate causal relationships between diseases, establishing themselves as reliable approaches for analysis using large‐scale GWAS data and circumventing the constraints inherent in observational studies. Certain effective combinations are investigated, and rational network‐based drug combination design strategies are established by measuring the link between diseases and drug networks, as well as between disease and disease networks [22]. In the initial phase, our research group integrated the network pharmacology method with the concept of multi‐target treatment. This approach enables modular analysis of intricate biological networks, breaking down extensive networks to identify specific modules. Subsequently, it simplifies and reduces the network's dimensionality, facilitating the measurement and integration of module intervention characteristics within the network [23, 24]. There is currently a dearth of studies assessing the correlation between MetS and rheumatic diseases. Our goal is to construct a disease–disease network, delineate modules, elucidate the distribution of these network modules, analyse potential mechanisms underlying their plausible correlation and contribute fresh insights and understanding to the treatment of rheumatic diseases. This will be achieved through an investigation into the correlation and causality between MetS and rheumatic diseases.

## 2 Methods

### 2.1 GWAS Data Source

MetS‐GWAS data were sourced from Van Walree et al. [25] Access to the data is available through the CTG database (https://ctg.cncr.nl/software/summary_statistics). Data related to fasting blood glucose (FBG), waist circumference (WC), essential hypertension (HY), HDL cholesterol (HDLC) and triglycerides (TGs) were retrieved from the IEU Open GWAS database (https://gwas.mrcieu.ac.uk/). Additionally, aggregated GWAS data for alcoholic drinks per week and smoking initiation were also obtained from the same database. Rheumatic diseases, encompassing RA, AS, SLE, SS, OS and gout (GO), were investigated using GWAS data retrieved from the Finngen database (https://www.finngen.fi/fi). Specific details regarding the data employed are outlined in Table 1. It is important to note that all datasets utilised in this study were acquired from publicly accessible sources.

**TABLE 1:** The GWAS data source of metabolic syndrome and rheumatic diseases.

Exposure/outcome	GWAS ID	Sample size	Number of SNPs	Population	Consortium	Source
Metabolic syndrome	CF_MetS_results_adapt	461,920	2,251,359	European	CTG‐LAB	https://ctg.cncr.nl/software/summary_statistics/
Fasting blood glucose	ebi‐a‐GCST005186	58,074	2,599,409	European	NA	https://gwas.mrcieu.ac.uk/
Waist circumference	ukb‐b‐9405	462,166	9,851,867	European	MRC‐IEU	https://gwas.mrcieu.ac.uk/
Essential hypertension	ukb‐b‐12,493	463,010	9,851,867	European	MRC‐IEU	https://gwas.mrcieu.ac.uk/
HDL cholesterol	ieu‐b‐109	403,943	12,321,875	European	UK Biobank	https://gwas.mrcieu.ac.uk/
Triglycerides	ieu‐b‐111	441,016	12,321,875	European	UK Biobank	https://gwas.mrcieu.ac.uk/
Alcoholic drinks per week	ieu‐b‐73	335,394	11,887,865	European	GWAS and Sequencing Consortium of Alcohol and Nicotine use	https://gwas.mrcieu.ac.uk/
Smoking initiation	ieu‐b‐4877	607,291	11,802,365	European	GSCAN	https://gwas.mrcieu.ac.uk/
Rheumatoid arthritis	finn‐b‐M13_RHEUMA	232,501	16,380,169	European	NA	https://www.finngen.fi/en
Ankylosing spondylitis	finn‐b‐M13_ANKYLOSPON	251,394	16,380,022	European	NA	https://www.finngen.fi/en
Systemic lupus erythematosus	finn‐b‐SLE_NOS	260,279	16,380,466	European	NA	https://www.finngen.fi/en
Sicca syndrome	finn‐b‐M13_SJOGREN	254,956	16,380,454	European	NA	https://www.finngen.fi/en
Osteoporosis	finn‐b‐M13_OSTEOPOROSIS	332,020	16,380,452	European	NA	https://www.finngen.fi/en
Gout	finn‐b‐M13_GOUT	228,784	16,380,152	European	NA	https://www.finngen.fi/en

### 2.2 General Genetic Correlation Analysis

The LDSC v1.0.1 application was used to assess genetic correlation (*r*_g_) and heritability (*h* ^2^) of the association between MetS and various rheumatic diseases. In linkage disequilibrium score regression (LDSC) regression, the linkage disequilibrium (LD) score of the single‐nucleotide polymorphism (SNP) site is the dependent variable, and this value is the independent variable of the approach. A self‐defined statistic is consistent with the chi‐square distribution. Linear regression is used to fit the relationship between the LD score and chi‐square statistic to determine if any confounding variables affect the GWAS analysis outcomes. It can be proven that confounding factors are not solely responsible for the overall phenotypic correlation if the genetic association is both statistically and quantitatively significant [26, 27]. Lastly, the ‘forestploter’, ‘pheatmap’, ‘corrplot’ and ‘ggplot2’ packages are used to generate the results as heat maps, bar charts and correlation charts.

### 2.3 Local Genetic Correlation Analysis

Traditional global approaches could ignore situations where the overlapped information is limited to particular areas or has conflicting orientations at various loci since they only take into account the average *r*_g_ across the genome. Using local genomic areas, local analysis of variant association (LAVA) could identify shared genetic association regions between phenotypes. Furthermore, more complex and conditional genetic associations could be obtained by performing paired local *r*_g_ tests on genomic loci using multivariate genetic association analysis.

### 2.4 Cross‐Trait Meta‐Analysis

The cross‐phenotype association analysis (CPASSOC) method was employed for assessing effect estimation and standard error in GWAS precision measurements [28]. This analysis utilised the installation packages ‘dplyr’ and ‘data.table.’ The method aimed to scrutinise shared genetic variations between SNPs and two traits. Paired sample heterogeneity (S_Het_) was employed to segregate and amalgamate data pertaining to MetS and its phenotypes with various rheumatic diseases. Relevant SNPs were identified using PLINK function parameters: clump‐p1 5E‐8, clump‐p^2^ 1E‐5, clump‐r^2^ 0.2 and clump 500 kb. SNPs were considered significant if *p*_*(single trait)*_ was less than 1 × 10^−5^ and *p*_*CPASSOC*_ was less than 5 × 10^−8^. Subsequently, SNPs identified through CPASSOC underwent functional annotation, and gene mapping was carried out using the Ensembl Variant Effect Predictor (VEP) database (https://grch37.ensembl.org/) [29].

### 2.5 Colocalisation Analysis

Genetic loci associated with specific behaviours were incorporated as a result of the CPASSOC. The colocalisation method (COLOC) was subsequently utilised to ascertain whether the same genetic variation in the loci is responsible for both traits. Using a Bayesian approach, the posterior probabilities for five distinct hypotheses on the sharing of causal changes within a genomic region are calculated. Among them are the following hypotheses: H0 (no association), H1 or H2 (related to a particular characteristic), H3 (association with both traits, involving two distinct SNPs) and H4 (relationship with both traits, suggesting one shared SNP). If PPH4 is higher than 0.95, a locus is considered colocalised. This analysis was carried out with the ‘coloc’ software package.

### 2.6 Module Optimisation, Main Module Selection and Division

To further investigate the potential associations and mechanisms between different phenotypes of MetS and various rheumatic diseases at the network level, we established individual gene maps linking MetS, FBG, WC, HY, HDLC, TG and the different rheumatic diseases. The STRING database (https://string‐db.org/) was employed to construct protein–protein interaction (PPI) target networks for these associations. The resulting networks were visualised using Cytoscape (Version 3.6.1).

Three techniques were utilised to determine the functional modules of every group of PPI networks: molecular complex detection (MCODE), Markov cluster (MCL) and GLay. The module pharmacological computing platform (http://112.86.129.72:48081) was then utilised, employing the minimum network structure entropy method, to compare the efficacy of the three module partitioning approaches. This analysis aimed to determine the optimal method for module identification [30] and was calculated according to the following formula:

$$E=-\sum_{i=1}^{n}I_{i}\ln I_{i}$$

where *N* represents the total number of nodes within the network, and *I*_i_ denotes the importance of its node. Node importance is defined as the proportion of the connectivity of each node relative to the total connectivity across all nodes.

In line with prior research methodology, identification of main modules involves sorting and calculating module node strength, betweenness centrality and PageRank [31, 32, 33]. Employing these three methods to rank the primary and secondary relationships within each module, the module securing the top position in each group is regarded as the candidate main module. Consistency across the outcomes of the first module obtained through all three methods designates it as the main module. In cases of inconsistency, the module undergoes further scrutiny to assess its impact on the eigenpath length disturbance across the entire network of modules.

### 2.7 The Relationship Between MetS and Rheumatic Diseases Under the Network Module

Genes linked to MetS and its phenotypes create modules within the protein interaction set, with their network distance from the rheumatic disease module serving as an indicator of similarity [22]. The comparative analysis of network relationships between two disease targets, s_*AB*_, involves assessing the mean shortest distance within their respective groups and the mean shortest distance (d_*AB*_) between disease–disease target pairs:

$$S_{\mathrm{AB}}=\left\langle d_{\mathrm{AB}}\right\rangle -\frac{\left\langle d_{\mathrm{AA}}\right\rangle +\left\langle d_{\mathrm{BB}}\right\rangle }{2}$$

The *Z* value stands as a dependable metric for gauging the network proximity between MetS (*X*) and various rheumatic diseases (*Y*). The calculation of the shortest path length, denoted as *d* (*x*, *y*), between the MetS target (*x*) and the rheumatic disease protein (*y*) is determined by the following formula:

$$d\left(X,Y\right)=\frac{1}{\left\|Y\right\|}\sum_{y\in Y}\min_{x\in X}d\left(x,y\right)$$

$$z=\frac{d-\mu }{\sigma }$$

The two target topologies overlap when s_*AB*_ < 0. The two sets of targets are topologically separated when s_*AB*_ ≥ 0. The rheumatic disease proteins and the MetS disease targets are isolated from one another from a network‐based standpoint, and their corresponding *z* is ≥ 0. If not, *z* < 0.

### 2.8 Functional Enrichment Analysis

Before initiating the analysis, the following packages must be pre‐downloaded: ‘org.Hs.eg.db’, ‘dplyr’, ‘enrichplot’, ‘ggplot2’, ‘clusterProfiler’ and ‘DOSE’. Set threshold values at *p* < 0.05 and *Q* < 0.05. Subsequently, gene ontology (GO) function analysis and Kyoto Encyclopedia of Genes and Genomes (KEGG) pathway enrichment analysis were performed for the genes.

### 2.9 Clinical Analysis and Validation

Using the GEO database (https://www.ncbi.nlm.nih.gov/geo/) and multiple independent GEO validation datasets (GSE98895, GSE77298, GSE11886, GSE4588, GSE40611 and GSE13985), we evaluated the expression levels of shared genes across samples from the normal group, MetS and various rheumatic diseases. ROC curves were generated for the shared genes using the ‘ROC’ package, with the AUC calculated to assess their diagnostic power for both diseases. An AUC value greater than 0.5 was considered indicative of a statistically significant difference. Additionally, to determine the specific localisation of key target organs and clarify their cellular distribution, we utilised the Human Protein Atlas (HPA) database (https://www.proteinatlas.org/).

### 2.10 MR Analysis

#### 2.10.1 MR Hypothesis and Analysis of SNP Data

Rheumatic diseases were discovered as the consequence of the two‐sample MR analysis, along with MetS and its constituents as exposure variables. To screen for SNPs significantly related to MetS based on the *p* < 5 × 10^−8^ criterion, download the ‘TwoSampleMR’ package [34]. Second, the LD of relevant SNPs was assessed with respect to the following setting criteria: *r* ^2^ ≤ 0.001; clumping window, 10,000 kb.

#### 2.10.2 Two‐Sample Univariate and Multivariate MR Analysis

If each genetic variation adhered to the assumptions of the instrumental variables, the primary analysis method employed was the inverse‐variance weighted (IVW) method [35]. This method ensures a consistent estimation of the causal relationship between the exposure factors and the outcome. The Egger method and the weighted median method were also utilised for a reliable causal effect assessment of multiple genetic variants using aggregated GWAS data, operating under weak assumptions and relying on instrument‐strength assumptions independent of direct effects [36]. Causal effects are quantified as the slope of linear regression, while estimates of average pleiotropic effects of genetic variation are represented as intercepts. To identify and mitigate potential bias, the MR‐PRESSO method was applied to perform outlier detection and distortion tests [37].

Previous observational studies have demonstrated the significant risk factors for the development of rheumatic diseases, which include smoking and alcohol use [38]. To evaluate the direct causal effects of alcohol and tobacco consumption on the risk of rheumatic illnesses, a multivariate MR (MVMR) study was performed using the ‘Mendelian randomization’ package.

#### 2.10.3 Sensitivity Analysis

The study evaluated heterogeneity among individual SNP estimates utilising Cochran's *Q* statistic. In instances where heterogeneity was detected (Cochrane's *Q p* < 0.05), a random effects model was employed [39]. The investigation additionally explored the presence of confounding variables in the results of the horizontal pleiotropy test [40]. To assess the stability of effect size and pinpoint specific SNPs contributing significantly more to the association than others, a leave‐one‐out analysis was conducted.

**FIGURE 1:**
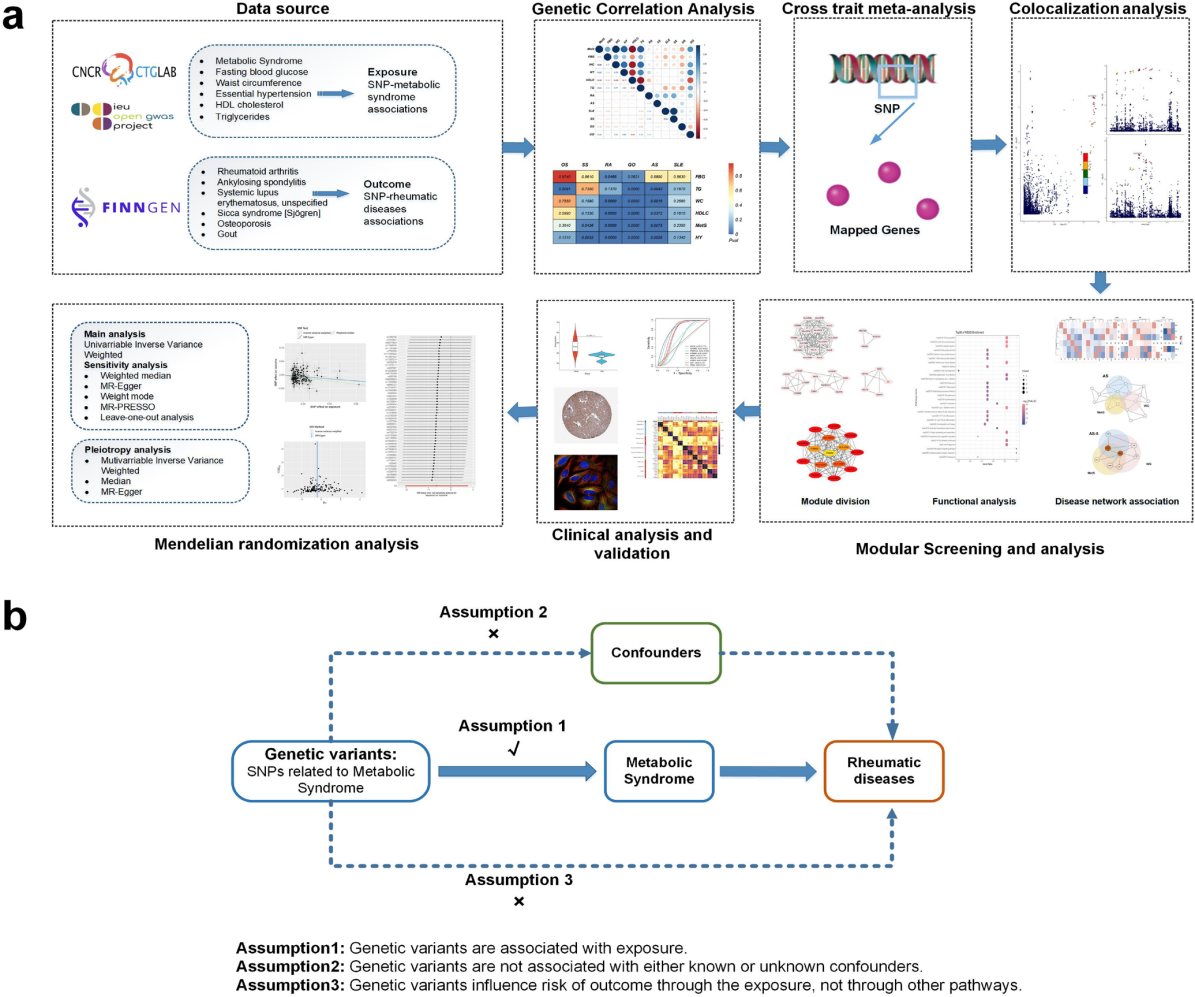
Overall study design. (a) Flow chart of this study. (b) The principles and main design of Mendelian randomisation study.

The study used R (Version 4.1.3) for all statistical calculations. A double‐tailed *p* < 0.05 was used to indicate statistical significance. Figure 1a depicts the precise operational procedures of this study, and Figure 1b depicts the precise procedures of MR hypothesis.

## 3 Results

### 3.1 Genetic Association Between MetS and Rheumatic Diseases

**FIGURE 2:**
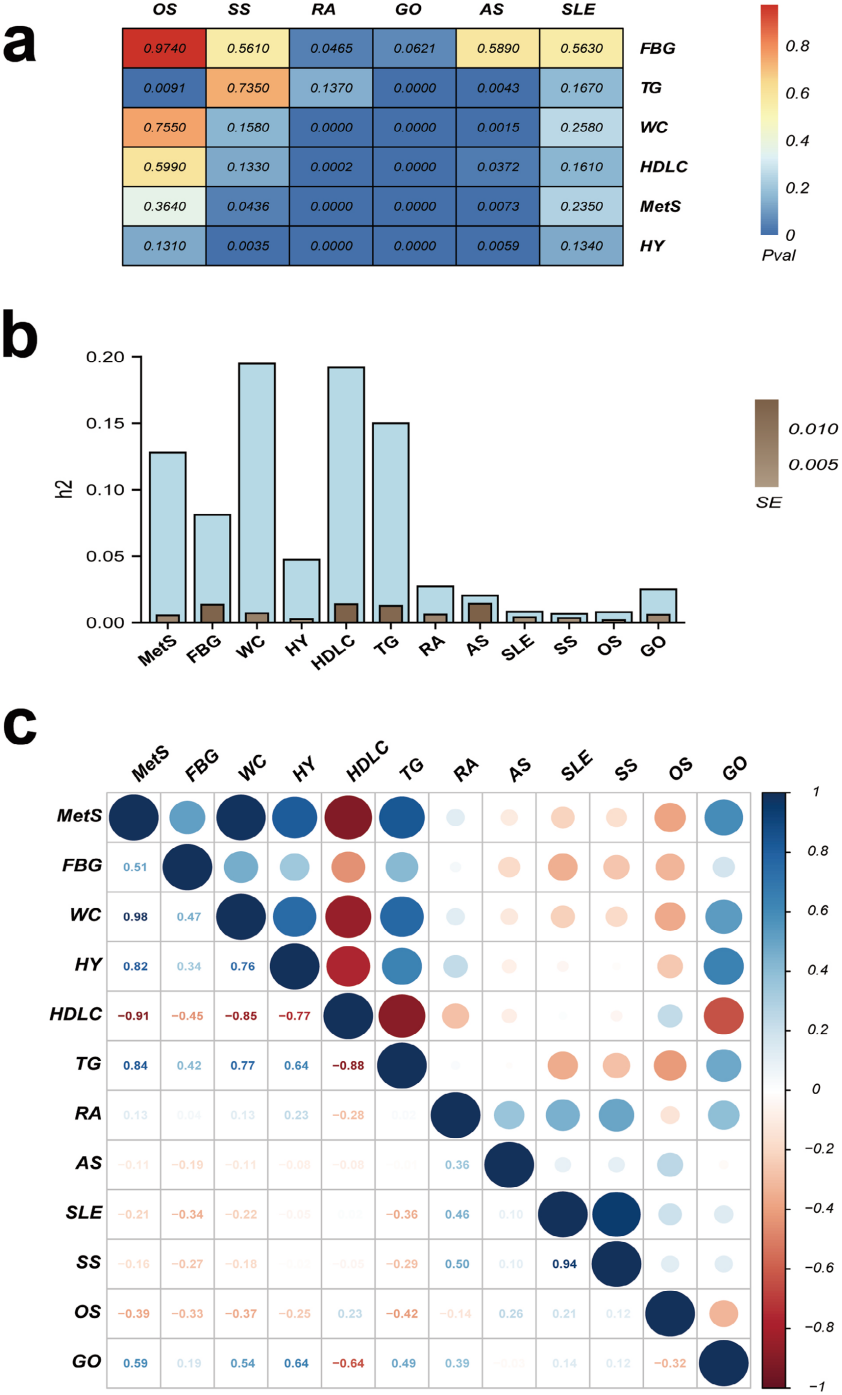
Heritability of metabolic syndrome and rheumatic diseases and their genetic correlation estimated using LDSC. (a) *p* value. (b) liability‐scale heritability *h* ^2^ (SE). (c) genetic correlation *r*_*g*_.

**FIGURE 3:**
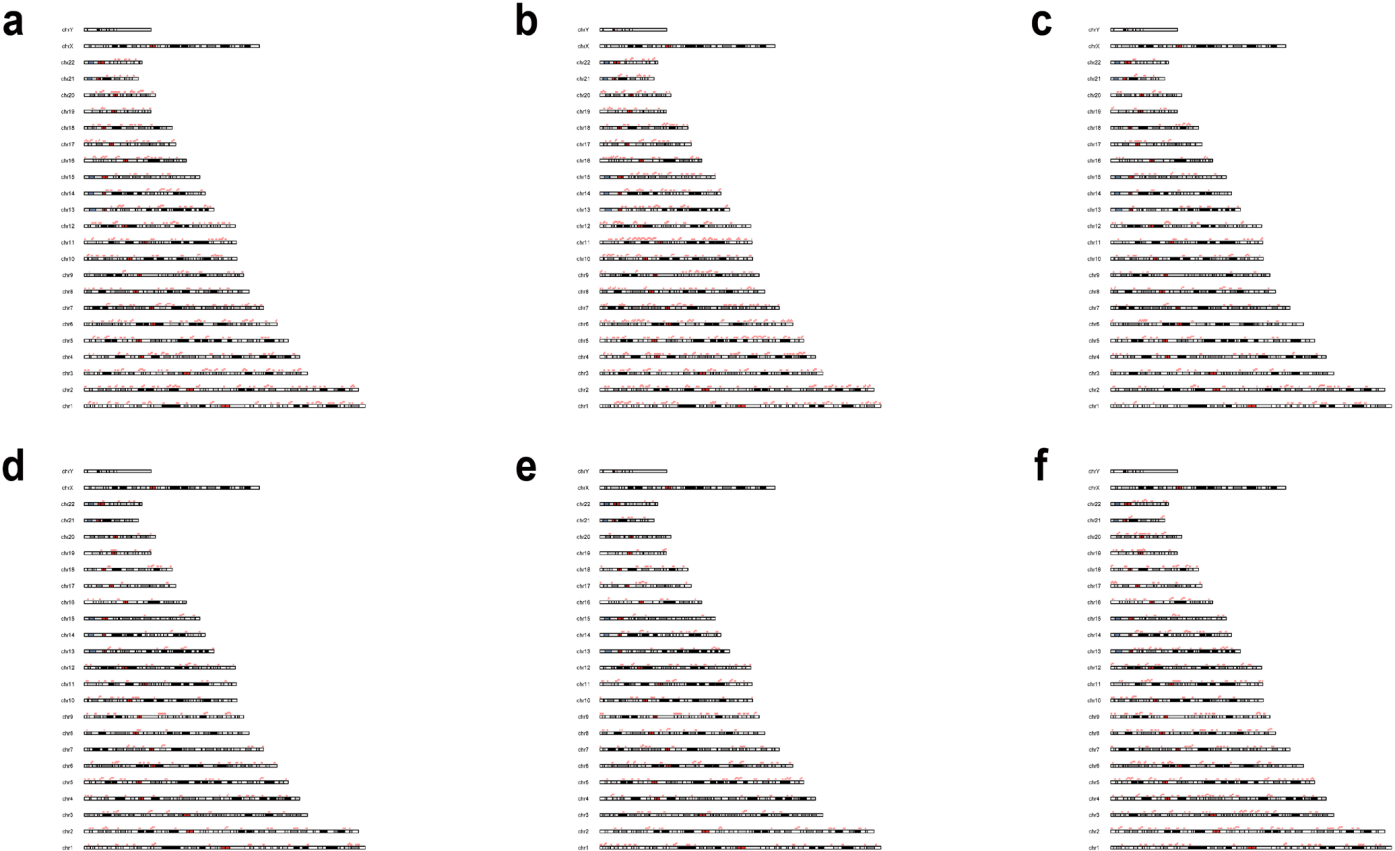
Analysis of the distribution of peak chromosomes in relation to local genetic correlations between metabolic syndrome and rheumatic diseases (*p* < 0.05). (a) RA. (b) AS. (c) SLE. (d) SS. (e) OS. (f) GO.

LDSC determines the heritability *h* ^2^ values: MetS (0.128, SE = 0.0054), FBG (0.0812, SE = 0.0135), WC (0.1950, SE = 0.0071), HY (0.0473, SE = 0.0025), HDLC (0.1920, SE = 0.0138), TG (0.1500, S E = 0.0126), RA (0.0272, SE = 0.0060), AS (0.0203, SE = 0.0141), SLE (0.0082, SE = 0.0039), SS (0.0066, SE = 0.0034), OS (0.0078, SE = 0.0018) and GO (0.0251, SE = 0.0058). There was a strong positive genetic correlation between MetS and RA, AS, SS and GO (*r*_*g(RA)*_ = 0.193, *p* = 8.8E‐09; *r*_*g(AS)*_ = 0.088, *p* = 0.0073; *r*_*g(SS)*_ = 0.106, *p* = 0.0436; *r*_*g(GO)*_ = 0.408, *p* = 7.44E‐32), especially the relation between HY and GO (*r*_*g*_ = 0.422, *p* = 1.38E‐17) (Figure 2). We have identified local correlations between MetS and different rheumatic diseases in LAVA. The most significant locus among them, with a *p* value of 3.74E‐21, was between MetS and AS at chr: bp 6:24852275–25684587. The second significant locus, with a *p* value of 1.28E‐16, was discovered at chr: bp 6:30798168–31571218 (Figure 3). Furthermore, comprehensive data pertaining to the local genetic association analyses have been provided in Table S1.

### 3.2 Identify the Shared Risk SNPs Between MetS and Rheumatic Diseases

We identified a total of 24 index loci and 37 mapped genes shared between MetS and rheumatic diseases (RA: rs2857597, rs9267529, rs592229, rs2071540, rs7184597, rs2271881;mapped genes: PRRC2A, AIF1, LY6G5B, CSNK2B, GPANK1, AL670886.1, SKIV2L, NELFE, TAP1, PSMB8, PSMB9, RABEP2, RAB3A, MPV17L2; AS: rs3789224, rs2328893, rs806973, rs12189841, rs3094549, rs10947210, rs2857597, rs1077394, rs9267529, rs592229, rs204993, rs9268005, rs2282850, rs4711346, rs412392, rs12205331; mapped genes: DCDC2, SLC17A4, HMGN4, OR5V1, OR12D3, PRRC2A, AIF1, BX511262.2, BAG6, PRRC2A, BX511262.2, LY6G5B, CSNK2B, GPANK1, AL670886.1, CR753842.1, BX511262.2, SKIV2L, NELFE, GPSM3, PBX2, AGER, RNF5, TAPBP, RGL2, PFDN6, ANKS1A; SLE: rs10214468, rs644045, rs204993; mapped genes: SLC17A1, C2, AL662834.1, CR388219.1, CR759782.1, GPSM3, PBX2, AGER, RNF5; SS: rs644045, rs204993;mapped genes: C2, AL662834.1, CR388219.1, CR759782.1, GPSM3, PBX2, AGER, RNF5; GO: rs12753981, rs3739095, rs4666000; mapped genes: GTF3C2, ZNF512). For further details, refer to Table S2.

The most notable SNP for both MetS and RA is identified as rs7184597 (*p*_*CPASSOC*_ = 7.89E‐30; mapped gene: RABEP2). Furthermore, rs412392, rs644045 and rs3739095 were identified as the most significant SNPs between MetS and AS (*p*_*CPASSOC*_ = 3.50E‐16), SLE (*p*_*CPASSOC*_ = 2.51E‐06), SS (*p*_*CPASSOC*_ = 2.51E‐06) and GO (*p*_*CPASSOC*_ = 1.12E‐06). The mapped genes associated with these SNPs include C2, AL662834.1, CR388219.1, CR759782.1 and GTF3C2. While no shared sites were identified between MetS and OS, notable shared sites emerged between WC, HY, HDLC and TG with OS: rs2760975, rs6108784 and rs56324610.

### 3.3 Identification of Mapped Genes

**FIGURE 4:**
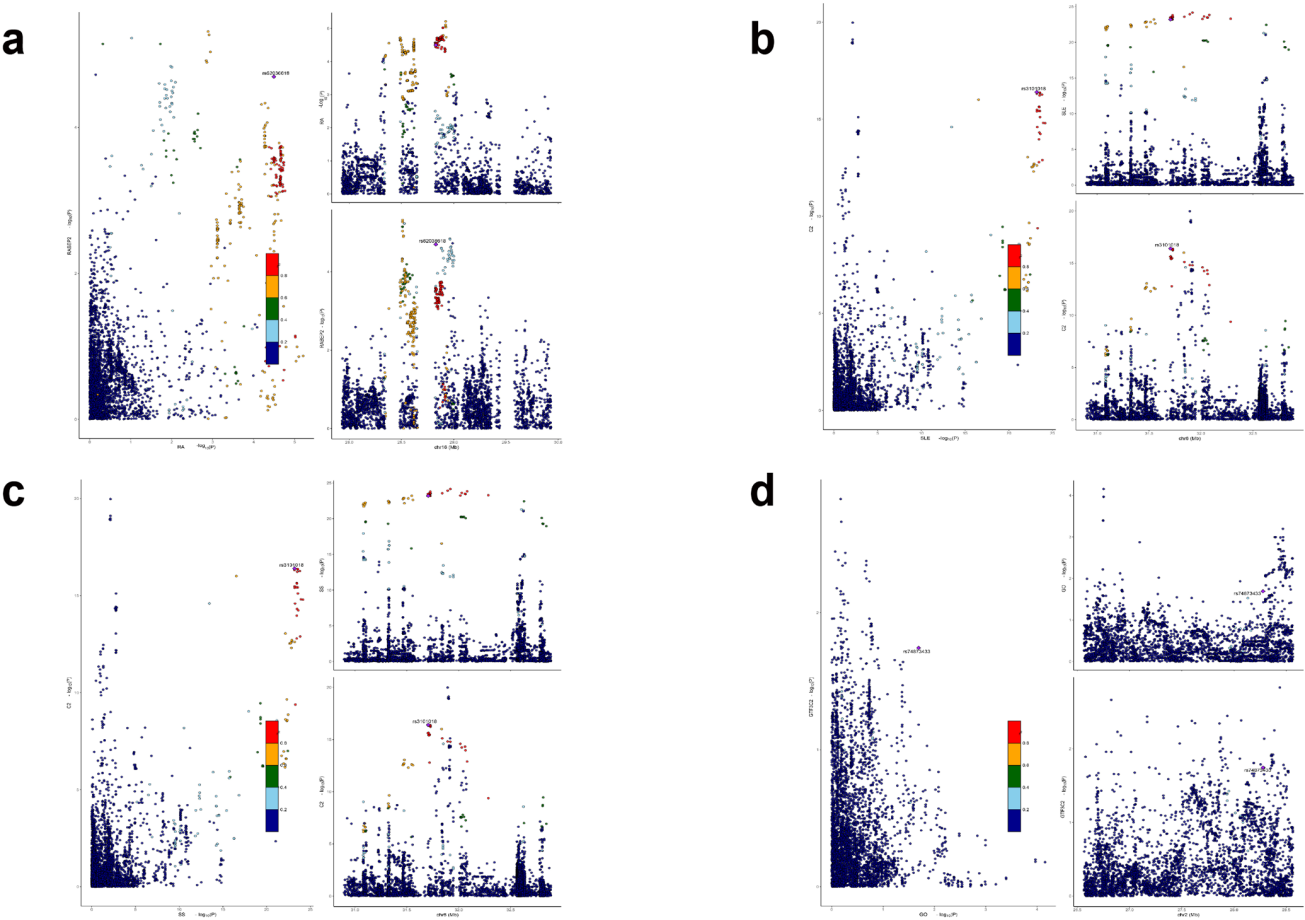
Colocalisation analysis. (a) Colocalisation analysis of RABEP2 with MetS and RA. (b) colocalisation analysis of C2 with MetS and SLE. (c) colocalisation analysis of C2 with MetS and SS. (d) colocalisation analysis of GTF3C2 with MetS and GO. The LD between the variations and the most significant SNPs is indicated by the *r* ^2^ value.

The colocalisation analysis of shared genetic loci between MetS and rheumatic diseases revealed 29 loci with PPH4 values exceeding 95%. Notably, rs2271881 (PPH4 = 0.9629) was identified as co‐mapped between MetS and RA at MPV17L2, corroborating findings from the cross‐trait meta‐analysis. The most significant SNPs, rs34434863, rs147725790 and rs45457097, were found within multiple genes, including AGER, LY6G5B, BAG6, CSNK2B, GPSM3, PBX2, PSMB8, PSMB9 and AIF1. In the colocalisation analysis of disease phenotypes and rheumatic diseases, 47, 22, 28 and 23 loci colocalised with WC, HY, HDLC and TG, respectively, all with PPH4 values greater than 95%. Among these loci, rs3748655 (PPH4 = 0.9999) was notably co‐mapped between WC and RA at MOV10 in alignment with results from the cross‐trait meta‐analysis. Key loci such as rs147725790, rs45457097, rs149580707, rs6472, rs2071467, rs9276815, rs1144709 and rs2269426 were found in genes, including LY6G5B, TAP1, PSMB8, PSMB9, SKIV2L, HLA‐DMB and HLA‐DOA. Additionally, our analysis revealed that RABEP2 (shared between MetS and RA), C2 (shared between MetS and SLE/SS) and GTF3C2 (shared between MetS and GO) were the most significant shared genes in the cross‐trait meta‐analysis. However, the PPH4 values for the loci mapped by these shared genes, rs62036618, rs3101018 and rs74873433, were all below 0.95, suggesting no clear genetic correlation between these shared genes and rheumatic diseases (Figure 4). Previous observational studies have indicated that these shared genes may play a role in the pathogenesis of both MetS and rheumatic diseases. Therefore, it is plausible that these observational findings were influenced by confounding factors such as environmental influences or acquired genetic variations. These findings suggest that shared genes play a role in the pathogenesis of MetS and various rheumatic diseases. The full results of the colocalisation analysis are available in Table S3.

### 3.4 Colocalisation Mapped Genes May Be Valuable Targets for Diseases Treatment

#### 3.4.1 GO and KEGG Analysis

**FIGURE 5:**
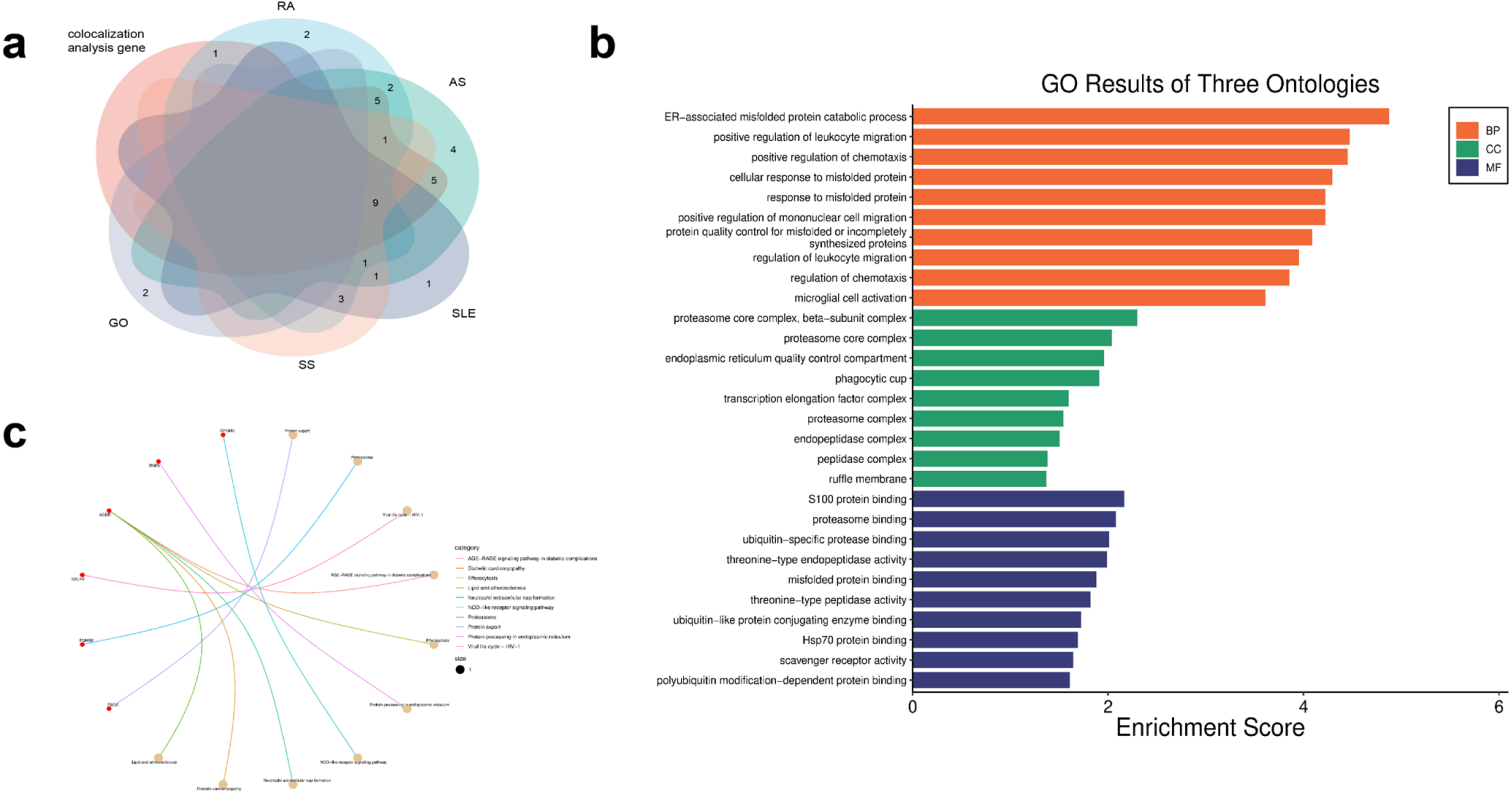
Identification and functional analysis of shared mapping genes. (a) The Venn diagram shows that rheumatic diseases and colocalisation‐mapped genes connect. (b, c) GO and KEGG analysis of the genes identified for colocalisation mapping.

To identify potential therapeutic targets for the treatment of multiple diseases, we investigated the shared genes between MetS and rheumatic diseases. Through colocalisation analysis, we found colocalisation‐mapped genes, including BAG6, GPSM3, PRRC2A, PSMB8, RNF5, NELFE, AGER, PBX2 and AIF1, which are common to RA, AS, SLE and SS (Figure 5a). Therapies for rheumatic diseases may include targeting these genes. GO analysis indicated that biological processes such as positive regulation of endoplasmic reticulum–associated misfolded protein catabolic process, glial cell activation, inflammatory response, cytokines, cell proliferation, synaptic transmission, protein–enzyme binding and the proteasome core complex may influence MetS and rheumatic diseases through these mapped genes (Figure 5b). Additionally, KEGG pathway analysis revealed significant enrichment in pathways related to glucose and lipid metabolism, AGE‐RAGE signalling in diabetic complications, NOD‐like signalling, viral cycles and neurodegenerative processes among the colocalisation=mapped genes (Figure 5c).

#### 3.4.2 Validation of Clinical Sample Expressions

**FIGURE 6:**
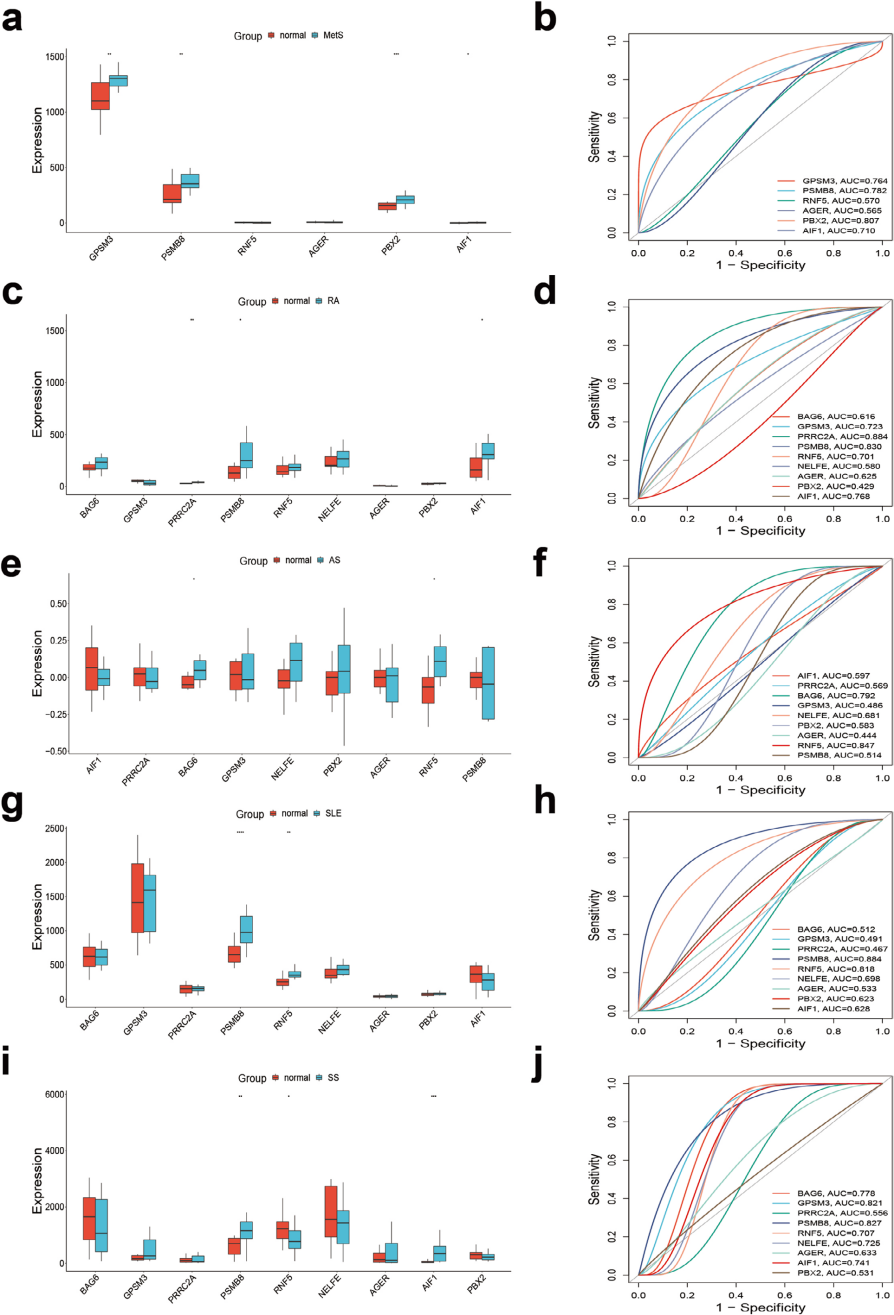
Validate the colocalisation‐mapped genes in GEO databases. (a, b) Gene expression levels in the normal and MetS groups, as well as the ROC curve for the MetS dataset's colocalisation‐mapped genes. (c, d) Gene expression levels in the normal and RA groups, as well as the ROC curve for the RA dataset's colocalisation‐mapped genes. (e, f) Gene expression levels in the normal and AS groups, as well as the ROC curve for the AS dataset's colocalisation‐mapped genes. (g, h) Gene expression levels in the normal and SLE groups, as well as the ROC curve for the SLE dataset's colocalisation‐mapped genes. (i, j) Gene expression levels in the normal and SS groups, as well as the ROC curve for the SS dataset's colocalisation‐mapped genes. *p < 0.05, **p < 0.01 and ***p < 0.001.

We selected the datasets GSE98895, GSE77298, GSE11886, GSE4588 and GSE40611 to verify the colocalisation‐mapped genes. Boxplot analysis revealed that GPSM3, PSMB8, PBX2 and AIF1 were significantly highly expressed in the MetS group (Figure 6a). In the RA group, PRRC2A, PSMB8 and AIF1 showed high expression levels (Figure 6c). For the AS group, BAG6 and RNF5 were notably elevated (Figure 6e), while PSMB8 and RNF5 were highly expressed in the SLE group (Figure 6g). In the SS group, PSMB8 and AIF1 exhibited high expression, whereas RNF5 was significantly downregulated (Figure 6i). All differences were statistically significant (*p* < 0.05).

#### 3.4.3 ROC Analysis

To evaluate the diagnostic value of the shared genes identified in this study, we constructed ROC curves and calculated the AUC values for the GEO dataset concerning MetS and the rheumatic diseases RA, AS, SLE and SS. The results indicated that PBX2 exhibited the highest diagnostic value for MetS, with an AUC of 0.807 (Figure 6b), while AGER demonstrated the lowest diagnostic value for both MetS and AS, with AUCs of 0.565 and 0.444, respectively (Figure 6f). Notably, the diagnostic value of PBX2 in RA and SS was low (AUC_*(RA)*_ = 0.429; AUC_*(SS)*_ = 0.531) (Figure 6d). PSMB8 showed the highest diagnostic accuracy for SLE and SS, with AUCs of 0.884 and 0.827, respectively (Figure 6h,j). PRRC2A had the highest diagnostic value for RA, achieving an AUC of 0.884; however, its AUC for diagnosing SLE was only 0.467. These shared genes demonstrate good predictive accuracy as potential biomarkers for MetS and rheumatic diseases.

### 3.5 The PPI Network Indicates an Association Between MetS, Phenotypic and Rheumatic Illnesses

#### 3.5.1 Module Division and Optimisation

**FIGURE 7:**
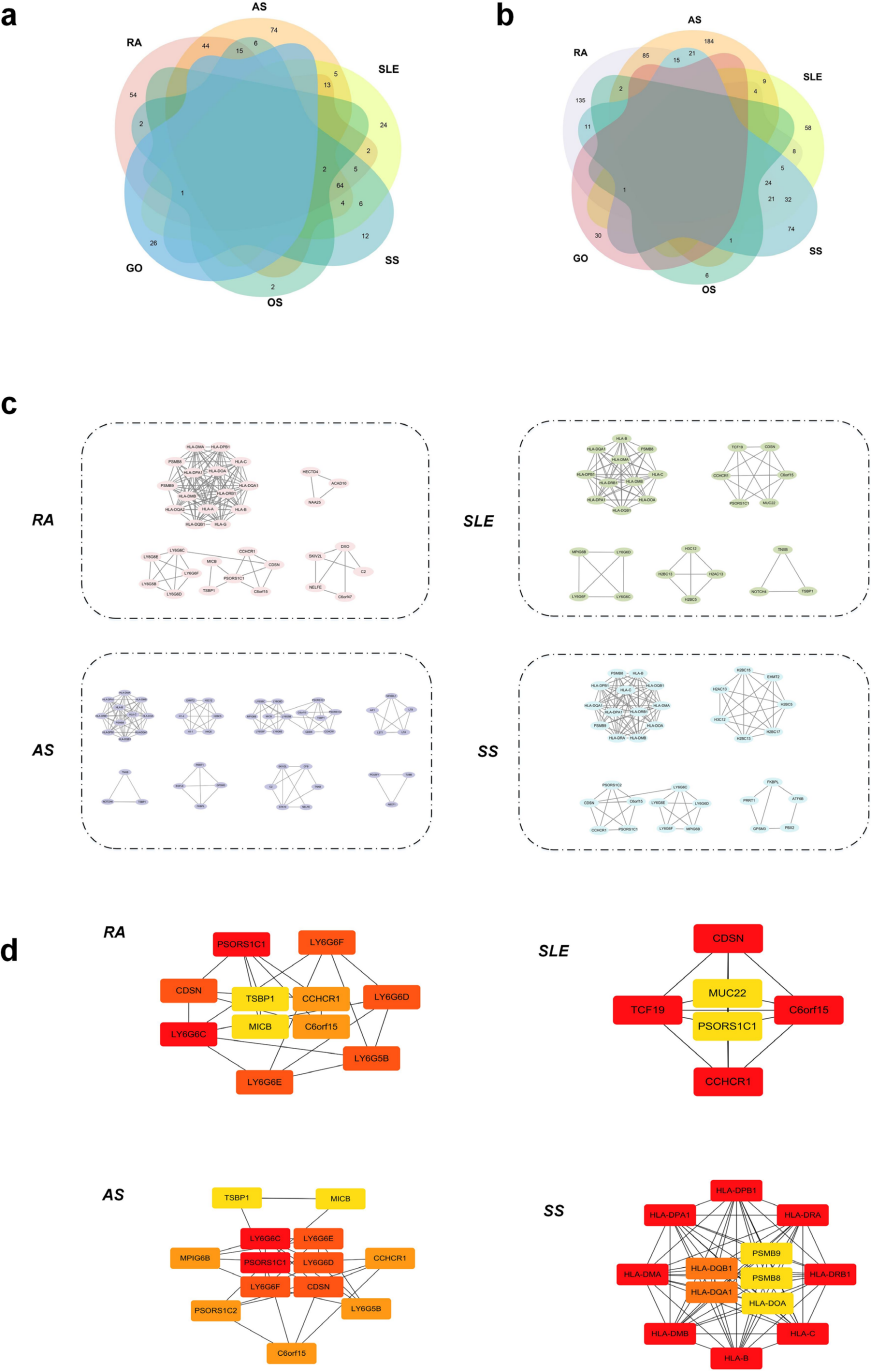
The PPI network was divided, identified and optimised based on module pharmacology. (a) The Venn diagram reflects the intersection of mapping genes for various rheumatic diseases. (b) The intersection of SNPs in various rheumatic diseases. (c) The MCODE algorithm divides rheumatic diseases into modules. (d) Screening of major modules of rheumatic diseases, divided by colours according to degree values.

In the previously discussed analysis of phenotypes characteristic of MetS and rheumatic diseases, WC and several rheumatic diseases collectively share 79 index loci, encompassing a total of 90 genes. HY shares 33 index loci, corresponding to 34 mapped genes. Additionally, HDLC and TG exhibit shared index loci of 317 and 379, respectively, mapping to 226 and 140 genes (Figure 7a,b). Nevertheless, no shared index site exists between FBG and rheumatic diseases. Network structure entropy serves as an effective descriptor for the disorder within scale‐free networks. The identification of functional modules from extensive networks involves a reduction in entropy, where a lower entropy indicates greater system stability. The calculated entropy values for each process are detailed in Table 2. Following the principle of minimum entropy, the MCODE algorithm is chosen as the method for module partitioning. RA, AS, SLE and SS were segmented into 4, 8, 5 and 4 modules, respectively (Figure 7c). Notably, Module 2 in RA, Module 3 in AS, Module 2 in SLE and Module 1 in SS were deemed major modules (Figure 7d). The genes allocated to these modules may potentially represent significant contributors to the impact of MetS and its phenotypes on RA, AS, SLE and SS.

**TABLE 2:** Comparison of three types of module division of target network (entropy).

	MCODE	MCL	GLay
Rheumatoid arthritis	3.288	4.217	4.205
Ankylosing spondylitis	3.821	4.272	4.377
Systemic lupus erythematosus	3.184	3.800	3.859
Sicca syndrome	3.417	3.868	4.018
Osteoporosis	—	—	—
Gout	—	2.079	2.079

#### 3.5.2 Relationships Between Multiple Networks of Rheumatic Diseases and the MetS and Its Phenotypes

**FIGURE 8:**
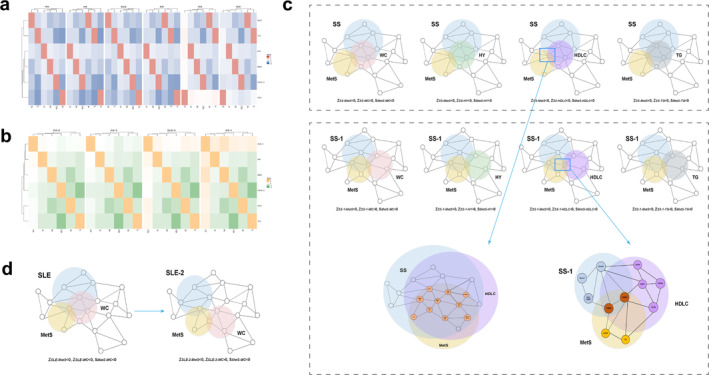
Network configurations of MetS–rheumatic disease combinations. (a, b) Association of MetS and its phenotypes with whole and major modules in rheumatic diseases. (c) Network‐based relationships between SS overall and major disease modules and MetS and component modules. (d) Genes were changed between MetS and SLE major modules.

Through the calculation of PPI network proximity between MetS targets and various rheumatic disease modules, it was observed that MetS targets exhibited a topological intersection with RA, AS, SLE, SS, OS and GO (*z* < 0) (Figure 8a). This topological overlap of MetS was consistent with the findings in the major disease modules RA‐2, AS‐3, SLE‐2 and SS‐1 (Figure 8b). It has been discovered that some of these genes (C2, BX511262.2, PRRC2A, BAG6, PBX2, GPSM3, NELFE and AIF1) are significant genetic targets shared by MetS and multiple rheumatic diseases. It is crucial to highlight that, concurrent with the identification of major modules, there exists a subset of genes like PSMB9 and PSMB8 that act as potential shared genes between SS‐1 and MetS, as well as with RA and SS (Figure 8c). In the context of SLE‐2 disease networks, the network proximity between SLE‐2, WC and HY were positive, while MetS, WC and HY were negative (*z* > 0; s_*AB*_ < 0), signifying topological distinctiveness between the two targets (Figure 8d). We provided Table S4 access to shared genes for MetS and their phenotypes associated with the main modules of rheumatic diseases.

#### 3.5.3 Biological Mechanisms and Validation of MetS and Phenotypes in Relation to Rheumatic Diseases

**FIGURE 9:**
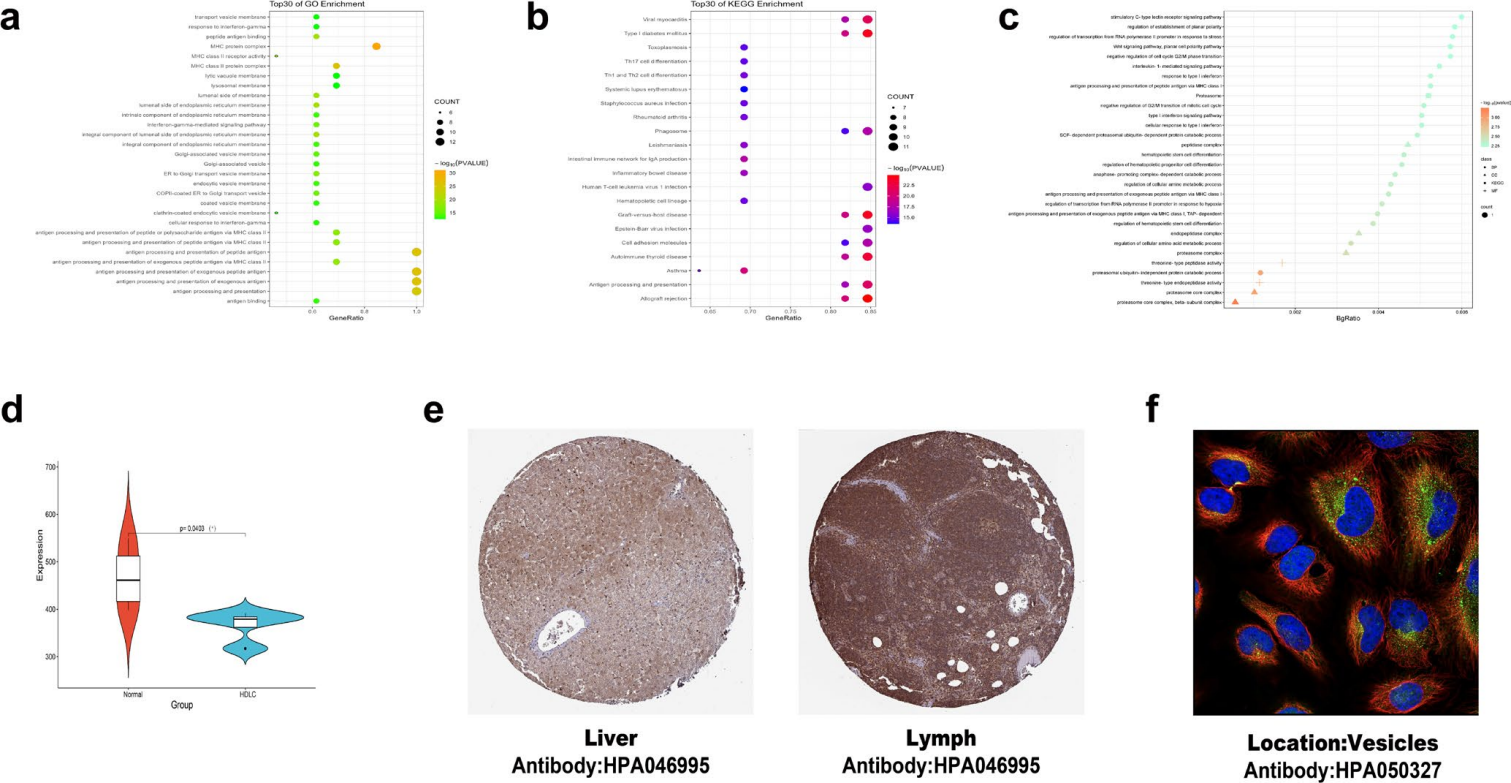
The biological mechanisms underlying rheumatic diseases and the verification of MetS and phenotypic. (a) GO functional analysis of rheumatic diseases (Top 30). (b) KEGG analysis of a variety of rheumatic diseases (Top 30). (c) GO and KEGG analysis of PSMB8. (d) PSMB8 expression levels in the normal and hyperlipidemia groups. (e) The IHC of PSMB8 in liver and lymph tissues. (f) The specific spatial distribution of PSMB8 in cells. *p < 0.05.

MetS's major phenotypes, mapped genes and the main modules of rheumatic diseases were analysed separately. GO analysis discovered that these genes were primarily enriched in biological processes related to the MHC protein complex, antigen processing and presentation and transport within the Golgi, endoplasmic reticulum and lysosomes (Figure 9a). KEGG analysis found strong correlations with a range of viruses, respiratory ailments and cardiovascular diseases in addition to involvement in IL‐17, Th1/Th2 and other immune signalling pathways (Figure 9b). Interestingly, the results showed that PSMB8 is essential to the main MetS and SS modules, especially for the production of the HDLC phenotype. This gene played a significant role in many biological processes, such as the creation of proteins and enzymes, transport, the processing and presentation of antigens, immunological response, cell cycle regulation, glucose and lipid metabolism and oxidative stress, according to functional enrichment analysis. Furthermore, Wnt, IL‐1, interferon and NF‐κB were only a few of the important signalling pathways that PSMB8 was strongly linked to (Figure 9c). We analysed the GSE13985 dataset to confirm the expression level of PSMB8 in the HDLC phenotype (Figure 9d). The results indicated that patients with hyperlipidaemia had significantly lower levels of HDLC and PSMB8 expression than the normal group, with a statistically significant difference (*p* < 0.05).

**FIGURE 10:**
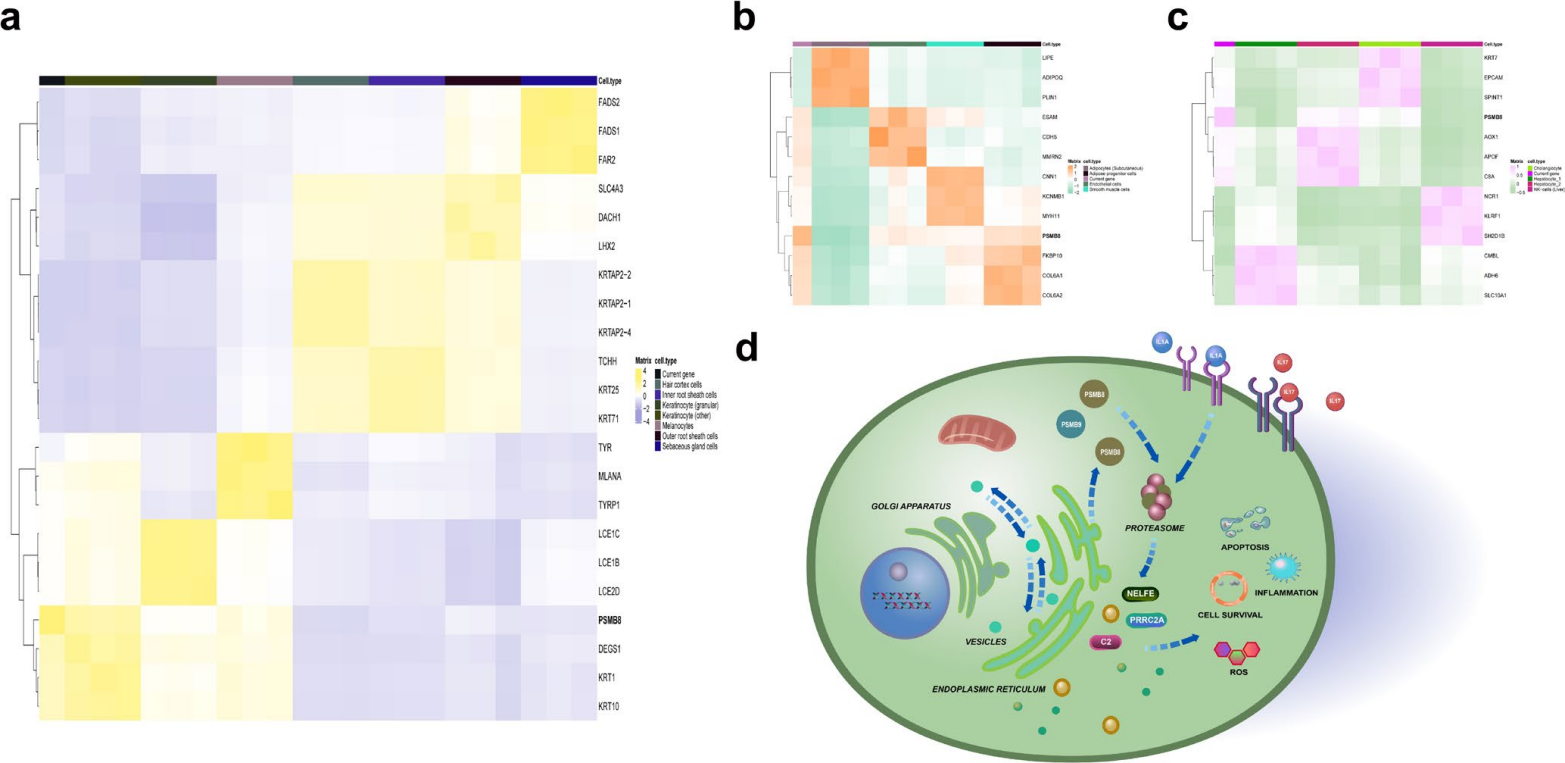
An association analysis of PSMB8 with prevalent biomarkers in skin (a), subcutaneous adipose tissue (b) and liver (c), along with a schematic representation of potential mechanisms of action (d).

The use of the HPA database offers valuable insights for validating gene expression at both cellular and histological levels. PSMB8 was found to be highly expressed in cholangiocytes, with moderate expression in hepatocytes, germinal centre cells and non‐germinal centre cells, suggesting its potential role in liver metabolism and lymphatic functions (Figure 9e). Correlation analysis revealed a negative association between PSMB8 and both subcutaneous adipocytes and liver cells, whereas a positive correlation was observed with skin keratinocytes (Figure 10). Notably, PSMB8 is predominantly localised in vesicles, indicating its critical role in the storage and transport of protein and enzyme complexes (Figure 9f). These spatial expression patterns underscore the complex regulatory mechanisms and diverse functions of PSMB8 in liver and lymphatic cells, providing deeper insights into its cellular localisation and potential physiological roles.

### 3.6 MR Analysis

#### 3.6.1 MetS and Rheumatic Diseases

**FIGURE 11:**
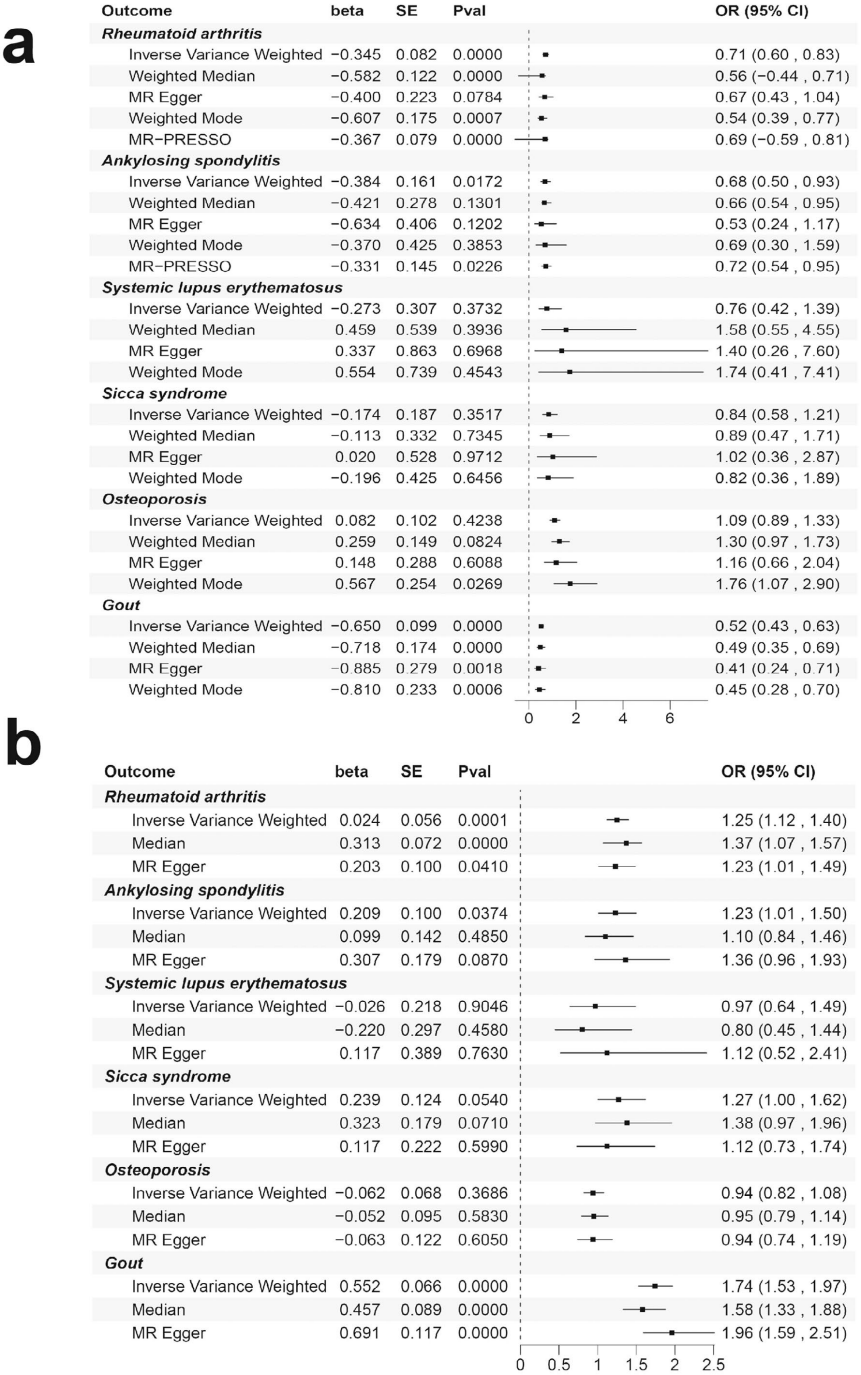
Causal effects of metabolic syndrome on rheumatic diseases estimated by UVMR (a) and MVMR (b) analysis.

**FIGURE 12:**
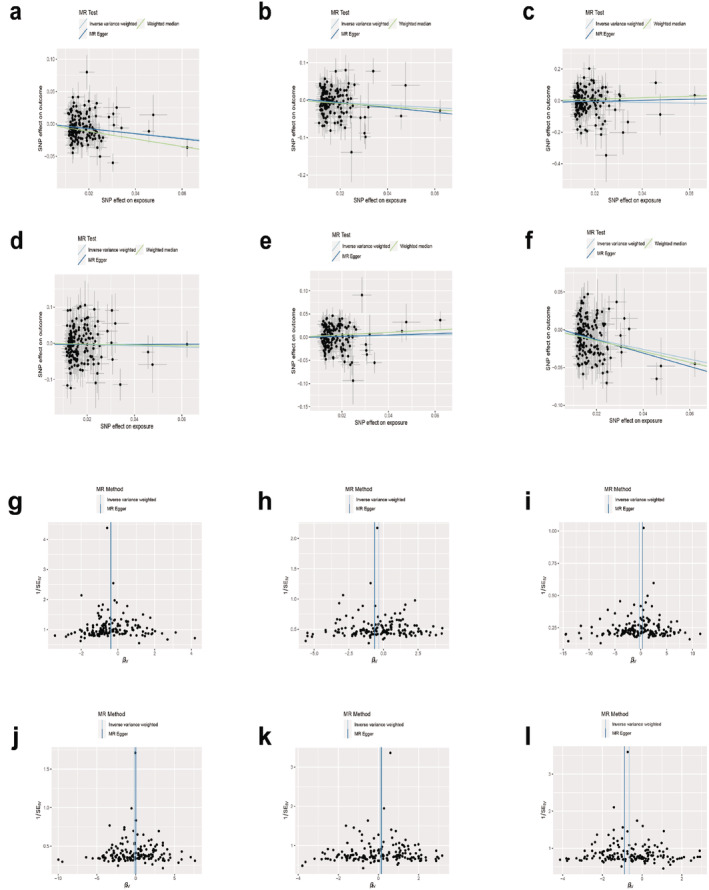
Scatter and funnel plots of metabolic syndrome with the risk of rheumatic diseases: RA (a, g), AS (b, h), SLE (c, i), SS (d, j), OS (e, k) and GO (f, l).

Univariable MR (UVMR) analysis showed that MetS was associated with RA, AS and GO (IVW OR_RA_ 0.7082, 95% CI: 0.6029–0.8319, *p* = 2.67E‐05; IVW OR_AS_ 0.6811, 95% CI: 0.4966–0.9341, *p* = 0.0172; IVW OR_GO_ 0.5222, 95% CI: 0.4298–0.9547, *p* = 6.30E‐11). The MR‐Egger method indicated no statistically significant difference between the zero intercept of the IVW method and other diseases, with the exception of GO. The weighted median methods for diseases, excluding RA and GO, demonstrated no statistical difference between the intercept and the zero intercept of IVW. Similarly, the weighted mode method for diseases, excluding RA, OS and GO, exhibited no statistical difference between the intercept and the zero intercept of IVW. The MR‐PRESSO test identified outliers as rs12608504, rs7581217, rs2066295 and rs12608504. Following their removal, there was no significant change (OR_RA_ 0.6929, 95% CI 0.5931–0.6345, *p* = 3.85E‐06; OR_AS_ 0.7185, 95% CI 0.5408–0.9547, *p* = 0.0226). There was no evidence of horizontal pleiotropy between the instrumental factors and the outcomes (*p* = 0.3677). The data showed no evidence of weak instrumental variable bias (*F* = 54.38). There was no vulnerability to genetic variation, as demonstrated by the leave‐one‐out cross‐validation method. After adjusting for smoking and alcohol application, MVMR analysis revealed a substantial relationship between MetS and RA, AS and GO (IVW OR_RA_ 1.251, 95% CI: 1.1211–1.3963, *p* = 6.31E‐05; IVW OR_AS_ 1.2327, 95% CI: 1.0123–1.5010, *p* = 0.0374; IVW OR_GO_ 1.7362, 95% CI: 1.5268–1.9742, *p* = 3.94E‐17) (Figures 11 and 12 and Table S5).

#### 3.6.2 FBG and Rheumatic Diseases

Following the removal of LD, 22 SNPs meeting the specified conditions were retained. After excluding the four SNPs with palindromes, the remaining 18 SNPs were included in subsequent analyses. The outcomes of the UVMR analysis revealed a causal relationship between FBG and AS (IVW OR_AS_ 1.9048, 95% CI: 1.1343–3.1987, *p* = 0.0148). MR‐PRESSO did not identify any outliers. In the MVMR analysis, no association was observed between FBG and rheumatic diseases after adjusting for smoking. The funnel plot, scatter plot and correlation sensitivity analysis are shown in Figures S1 and S2 and Table S6.

#### 3.6.3 WC and Rheumatic Diseases

A total of 304 SNPs were included in the follow‐up analysis after the removal of LD, independence of outcome factors, palindromes and repetitions. The results of the UVMR analysis indicated significant causal associations between WC and RA, AS, OS and GO (IVW OR_RA_ 0.7318, 95% CI: 0.6378–0.8397, *p* = 8.60E‐06; IVW OR_AS_ 0.7464, 95% CI 0.5770–0.9657, *p* = 0.0260; IVW OR_OS_ 1.216, 95% CI: 1.0213–1.4487, *p* = 0.0211; IVW OR_GO_ 0.5546, 95% CI: 0.4468–0.6883, *p* = 8.84E‐08). MR‐PRESSO identified outliers as rs2302209, rs28366156, rs883403 and rs4148155. Following their removal, the results were reanalysed, but no significant changes occurred, and the heterogeneity persisted (IVW OR_RA_ 0.7157, 95% CI: 0.6307–0.8121, *p* = 2.13E‐07; IVW OR_GO_ 0.5200, 95% CI: 0.4459–0.6064, *p* = 7.69E‐17). The MVMR analysis demonstrated that after adjusting for alcohol consumption and smoking, the causal relationship remained consistent with the findings of the previous analysis (IVW OR_RA_ 0.7768, 95% CI: 0.6658–0.9064, *p* = 0.0013; IVW OR_OS_ 1.2650, 95% CI: 1.0431–1.5341, *p* = 0.0169; IVW OR_GO_ 0.5725, 95% CI: 0.4450–0.7364, *p* = 1.42E‐05). These data can be found in the Figures S3 and S4 and Table S7.

#### 3.6.4 HY and Rheumatic Diseases

The UVMR analysis revealed a correlation between HY and GO (IVW OR_GO_ 0.0771, 95% CI: 0.0137–0.4344, *p* = 0.0037). Several outliers were detected through MR‐PRESSO analysis, yet the MR results remained unaffected after their removal (IVW OR_GO_ 0.1172, 95% CI: 0.0241–0.5692, *p* = 0.0078). Notably, the heterogeneity in AS, SLE, SS and OS disappeared. No evidence of horizontal pleiotropy or vulnerability to genetic variation between instrumental variables and results was identified. In the MVMR analysis, after adjusting for alcohol consumption and smoking, the causal association between HY and RA increased (IVW OR_RA_ 0.1236, 95% CI: 0.0349–0.4379, *p* = 0.0012), while the association between HY and GO persisted (IVW OR_GO_ 0.0450, 95% CI: 0.0092–0.2209, *p* = 0.0001). We uploaded these data in the form of Figures S5 and S6 and Table S8.

#### 3.6.5 HDLC and Rheumatic Diseases

The UVMR analysis indicates a correlation between HDLC and GO (IVW OR_GO_ 1.1897, 95% CI: 1.0307–1.3732, *p* = 0.0177). A total of 12 outliers were detected in the MR‐PRESSO test. Despite their removal, there was no significant alteration in the MR analysis results, and the heterogeneity in RA, AS, OS and GO persisted. The MR‐Egger regression method identified horizontal pleiotropy between the instrumental variable and GO (*p* = 0.0026), a phenomenon not observed with other diseases. Data retention cross‐validation demonstrated that no single SNP was sensitive to variation. Notably, the causal estimates remained consistent with the MVMR analysis (IVW OR_GO_ 1.1445, 95% CI: 1.0016–1.3077, *p* = 0.0473). We provide Figures S7 and S8 and Table S9 with these data.

#### 3.6.6 TG and Rheumatic Diseases

The UVMR analysis unveiled a causal relationship between TG and SLE, OS and GO (IVW OR_SLE_ 1.6523, 95% CI: 1.0140–2.6926, *p* = 0.0438; IVW OR_OS_ 1.1759, 95% CI 1.0009–1.3814, *p* = 0.0487; IVW OR_GO_ 0.7054, 95% CI: 0.5960–0.834, *p* = 4.88E‐05). Multiple outliers were identified through MR‐PRESSO analysis, yet the results remained largely unchanged after their removal. However, the heterogeneity in RA, AS and GO persisted (*Q*_RA_ = 368.49, *p* = 6.10E‐06; *Q*_AS_ = 311.19, *p* = 0.0145; *Q*_GO_ = 388.73, *p* = 3.11E‐07). The MR‐Egger regression method detected horizontal pleiotropy between the instrumental variable and GO (*p* = 0.0177). Leave‐one‐out cross‐validation demonstrated that the data were not sensitive to genetic variation. In the MVMR analysis, a significant association between TG and OS and GO was observed after adjusting for alcohol consumption and smoking (IVW OR_OS_ 1.1591, 95% CI: 1.0445–1.2862, *p* = 0.0054; IVW OR_GO_ 0.7962, 95% CI: 0.7089–0.8943, *p* = 0.0001). However, the MVMR analysis did not reveal an association between TGs and SLE. These data are uploaded as Figures S9 and S10 and Table S10.

## 4 Discussion

In this study, we undertook a comprehensive genetic analysis employing aggregated statistics from GWAS to investigate the genetic overlap, network associations and causality between MetS and rheumatic diseases. The outcomes of the genetic correlation analysis align closely with current clinical trial findings [41, 42], with the most pronounced positive genetic correlation observed between HY and GO. A prospective clinical study revealed a 1.48‐fold increased likelihood of developing GO in individuals with high blood pressure compared to those with normal blood pressure (95% CI: 1.28–1.71) [43]. Importantly, these findings do not imply a causal relationship where high blood pressure directly leads to GO development but instead suggest a shared genetic basis between the two sets of disease traits. A substantial genetic connection between MetS and AS, finely localised on Chromosome 6, was discovered through additional local genetic association analysis. This indicates a complex interplay between genetic and metabolic factors linking MetS with various rheumatic diseases. Prolonged disruptions in glycolipid metabolism and elevated systemic arterial blood pressure emerge as significant factors influencing the future risk of rheumatic diseases. Metabolic intermediates of glycolysis not only supply energy but also function as signalling molecules that regulate immune responses. Glucose‐6‐phosphate, a pivotal intermediate in both glycolysis and the PPP, influences the metabolism and function of immune cells, contributing to immune dysregulation in rheumatic diseases [44]. Moreover, fluctuations in leptin levels, a key regulatory molecule in energy metabolism, impact the leptin signal transduction pathway, resulting in aberrant regulation of macrophages and fibroblasts and ultimately contributing to dysfunction in bones and joints [45, 46].

The CPASSOC analysis results reveal that the shared mapping genes among MetS, its phenotypes and various rheumatic diseases predominantly cluster in the endocrine, digestive and respiratory systems. The significance of these shared genes was further validated through the results of the colocalisation analysis. Given that rheumatic diseases are a subset of autoimmune disorders closely associated with specific organs and tissues, this concentration indicates a strong correlation. Notably, investigations highlight the role of RABEP2 as a substrate for glycogen synthase kinase GSK3β, influencing cell membranes, lysosomes and macroplastids via phosphorylation at Ser200 and Ser204 sites [47]. GSK3β is a critical regulator in various metabolic pathways. The balance between Akt and GSK3β may be upset by aberrant RABEP2 regulation, which would further affect mTORC1 activation. This aberrant activation increases the synthesis and release of lactic acid and nitric oxide, stimulates aerobic glycolysis and worsens the inflammatory response in RA and SLE. However, aberrant GSK3β phosphorylation and mTOR activation work together to promote excessive extracellular matrix deposition and accelerate the development of fibrosis in the synovial and cartilage tissues of AS [48, 49]. Furthermore, RABEP2 acts as a regulatory factor promoting Rab4‐dependent VEGFR2 transport, inhibiting the VEGFR2/PKC/ERK1‐2 signalling pathway, reducing Sph K1 translocation and effectively impeding synovial angiogenesis, thereby alleviating RA progression [50, 51]. Although literature on AL662834.1, CR388219.1 and CR759782.1 is limited, their confirmed roles in SLE and SS warrant further experimental validation.

**FIGURE 13:**
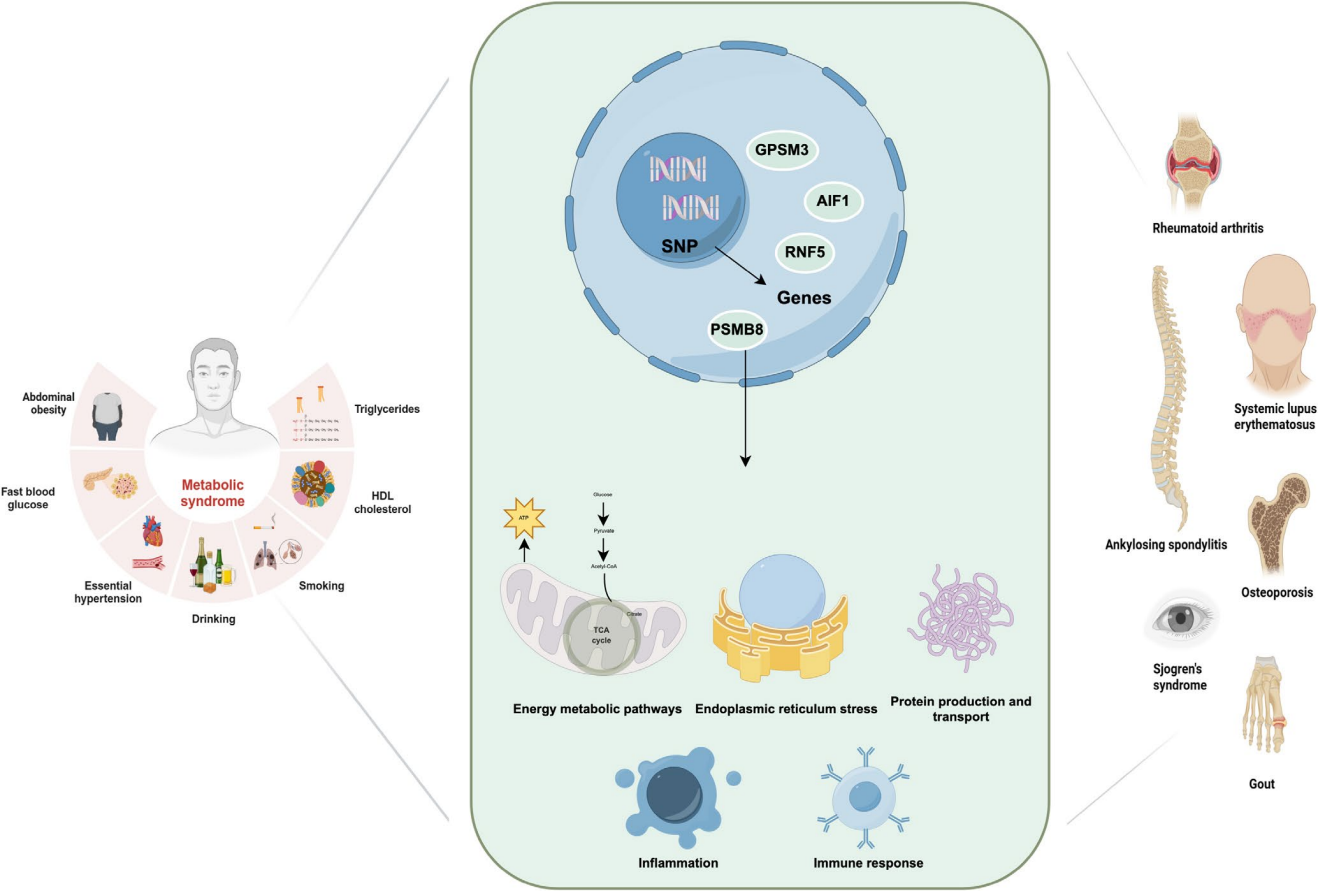
Schematic illustration of the possible mechanisms of MetS and rheumatic diseases.

With the advent of systems biology and network pharmacology, the paradigm of studying disease and drug mechanisms has transitioned from the traditional ‘single component, single target, single disease’ model to a more comprehensive ‘multi‐component, multi‐target, multi‐pathway’ approach [52]. The pathogenesis of diseases exhibits a modular foundation, and the interconnectedness of these modules facilitates the execution of multiple biological functions within the network [53]. Disease‐specific immune metabolism can be affected by both genetic and environmental variables. The major genes implicated are PSMB8, PSMB9, BAG6, PRRC2A, PBX2 and NELFE. These genes have a significant impact on the pathophysiology of numerous rheumatic diseases, particularly by regulating the mTOR pathway and immunometabolism signalling. The immunoproteasome components encoded by PSMB8 and PSMB9 are principally in charge of processing and presenting antigens from MHC Class I molecules. By regulating the PI3K/Akt/mTOR axis, they have been demonstrated to be involved in the processes of cell proliferation, migration and antigen presentation [54]. In disease conditions, mTOR activation propels immunopathology via a variety of routes. As a result, clinical symptoms such AS, SLE, RA, AS and SS may be directly connected to its modifications [55]. PSMB8 appears in vesicles and can activate mTORC1 when the branched‐chain amino acids, glutamine, kynurenine and histidine that pass through by vesicles reach the lysosomes. It additionally regulates the metabolic remodelling of mitochondrial DNA and the restoration of antioxidant capacity, which in turn controls cell growth, proliferation and metabolism. To respond to stress and preserve cell homeostasis, this connection is essential [56]. Overexpression of PSMB8 and PSMB9 in SLE and RA patients may aggravate the inflammatory response and tissue damage, increase the activation of the PI3K/Akt/mTOR and NF‐κB signalling pathways and enable the release of proinflammatory cytokines TNF‐α and IL‐6 [18, 57]. PSMB8 and PSMB9 mutations cause metabolic remodelling, including increased glycolysis and PPP. The generation of ROS directly triggers the mTORC1 signalling pathway. Furthermore, PSMB8 overexpression may worsen mTOR activation by impairing autophagy and depleting mitochondrial DNA, which would lead to the aberrant development of proinflammatory T cell and macrophage phenotypes in SLE and RA [17]. Proinflammatory T cell production in AS patients is influenced by both metabolic abnormalities and hyperactivation of the mTOR pathway. According to further GWAS findings, HLA‐B27 misfolding aggravates aberrant immune cell activation via the mTOR pathway, triggers ER stress and promotes PI3K‐Akt signalling [58]. Through its interactions with PSMB8/PSMB9, BAG6 may influence immunoproteasome function and control antigen presentation [59]. Through the activation of CD8^+^ T cells and additional disruption of the equilibrium of bone metabolism, abnormal antigen presentation can exacerbate AS. By disrupting APC–mediated antigen presentation, mutations in PRRC2A and NELFE in SS patients may intensify the disease's immunopathological response. By regulating MHC Class II antigen processing and immune‐related gene expression, PRRC2A stimulates B cell activation and autoantibody synthesis [60]. Additionally, PRRC2A mutations could enhance metabolic pathways for the TCA cycle and oxidative phosphorylation, increasing the accumulation of ROS and NADPH, triggering mTORC1 through metabolic pathways and further promoting the production of proinflammatory cytokines and the growth of pathologic T cell subsets through Th1/Th2 signalling [61, 62]. Notably, the function of NELFE and PBX2 in controlling transcription and antigen presentation enhances the possibility of mTORC1 activation's aftereffects. The chronic state of mTOR activation exacerbates the clinical signs of rheumatic diseases by generating pro‐inflammatory necrosis and the depletion of metabolic resources. Furthermore, it was verified that GPSM3 was associated with to particular SNPS (rs204989 and rs204991), and via causing neutrophil migration, GPSM3 was related to a lower risk of RA, AS, SLE, Type 1 diabetes and multiple sclerosis. Furthermore, we discovered that BX511262.2 might be significant in MetS and other multiple rheumatic diseases; nevertheless, additional research needs to be done. Consequently, these genes hold potential clinical value for the diagnosis and treatment of MetS and associated conditions such as AS, SS and RA [63, 64] (Figure 13). Therefore, research integrating these recently identified genes with the mTOR pathway advances our knowledge of the pathophysiology of rheumatic disorders and offers fresh concepts for mTOR signalling‐based targeted therapy.

In this MR study, we explored the impact of MetS on diverse rheumatic conditions, encompassing key MetS phenotypes, namely FBG, WC, HY, HDLC and TG. This comprehensive assessment aimed to elucidate the causal relationships involved. The MR analysis revealed that MetS heightened the risk of RA, AS and GO. Additionally, individual phenotypes of MetS—FBG, WC, HY, HDLC and TG—contributed to an increased risk of various rheumatic diseases. Even after adjusting for smoking and drinking behaviours using MVMR, MetS maintained a direct causal effect on RA, AS and GO. Unhealthy lifestyle factors, such as poor diet, inadequate sleep and lack of exercise, are known contributors to adverse health outcomes [65]. A cohort study underscored the correlation between elevated FBG levels and worsened outcomes, as evidenced by higher scores on the Kellgren–Lawrence scale, increased knee injuries and compromised Arthritis Outcome Score (KOOS). Multiple logistic regression analysis further revealed a significant negative correlation between FBG and KOOS (*B* = −0.448, *p* = 0.003) [66]. In another prospective, randomised, parallel clinical study, it was demonstrated that individuals with MetS are prone to exhibiting low urinary pH, alterations in CRP levels and increased susceptibility to GO attacks, particularly in cases exacerbated by chronic hyperglycaemia and hyperlipidaemia [67]. However, the mechanisms underpinning these associations remain poorly understood. Based on the findings from various analyses, we hypothesise that MetS influences the construction, generation, transport and expression of protein complexes through pathways such as glycolysis, PPP, the TCA cycle, inflammatory response, immune response and endoplasmic reticulum stress. The inflammatory response of RA and AS can be exacerbated by changes in the amounts of metabolic intermediates of the TCA cycle, which can directly control the metabolic status of immune cells and activate pro‐inflammatory macrophages [68]. The metabolic ability of antigen‐presenting cells is further compromised by abnormalities of glycolysis and PPP, which leads to immunological assaults in SLE by disrupting immune tolerance and impairing antigen presentation. When PPP malfunctions in MetS, the body produces less NADPH and its antioxidant activity is decreased. This results in an excessive build‐up of ROS and further damage to joint tissues [69]. By affecting the downstream key factor mTOR, a multi‐omics sequencing study has found that a number of factors associated with inflammation, oxidative stress and autophagy could regulate the cellular inflammatory response, preserve intestinal flora and cellular metabolic homeostasis and control immune function [70]. In conclusion, the mTOR signalling pathway is the primary connection between MetS and rheumatic disorders, and MetS may be a significant risk factor for rheumatic conditions like RA, AS and GO. In addition to contributing to immunological and inflammatory disorders, its aberrant activation also increases the correlation between diseases via altering metabolism. Future research, particularly focusing on relevant basic experimental investigations and drug development, may find valuable avenues in exploring these connections further.

## 5 Conclusion

By identifying shared loci, delving into modular division and providing strong evidence of genetic correlations, this research has explored the connections between MetS and rheumatic diseases. These findings strengthen the understanding of observational relationships and offer solid evidence in favour of causal inferences.

## Author Contributions

**Yinli Shi:** conceptualization (equal), methodology (equal), visualization (equal), writing – original draft (equal). **Shuang Guan:** data curation (equal), formal analysis (equal). **Xi Liu:** data curation (equal), visualization (equal). **Hongjun Zhai:** data curation (equal), software (equal). **Yingying Zhang:** methodology (equal), software (equal), validation (equal). **Jun Liu:** supervision (equal), validation (equal), writing – review and editing (equal). **Weibin Yang:** methodology (equal), supervision (equal), writing – review and editing (equal). **Zhong Wang:** funding acquisition (equal), resources (equal), supervision (equal), writing – review and editing (equal).

## Ethics Statement

The authors have nothing to report.

## Consent

The authors have nothing to report.

## Conflicts of Interest

The authors declare no conflicts of interest.

## Supporting information


Figures S1–S10
Tables S1–S10
Data S1

## Data Availability

The data that support the findings of this study are openly available in the IEU Open GWAS database (https://gwas.mrcieu.ac.uk/) and the Finngen database (https://www.finngen.fi/fi).
