# Supplementary figures and images for: Genetic Commonalities Between Metabolic Syndrome and Rheumatic Diseases Through Disease Interactome Modules
Source: J Cell Mol Med. 2025 Jan 9;29(1):e70329. doi: 10.1111/jcmm.70329 (PMC11717667; doi:10.1111/jcmm.70329)

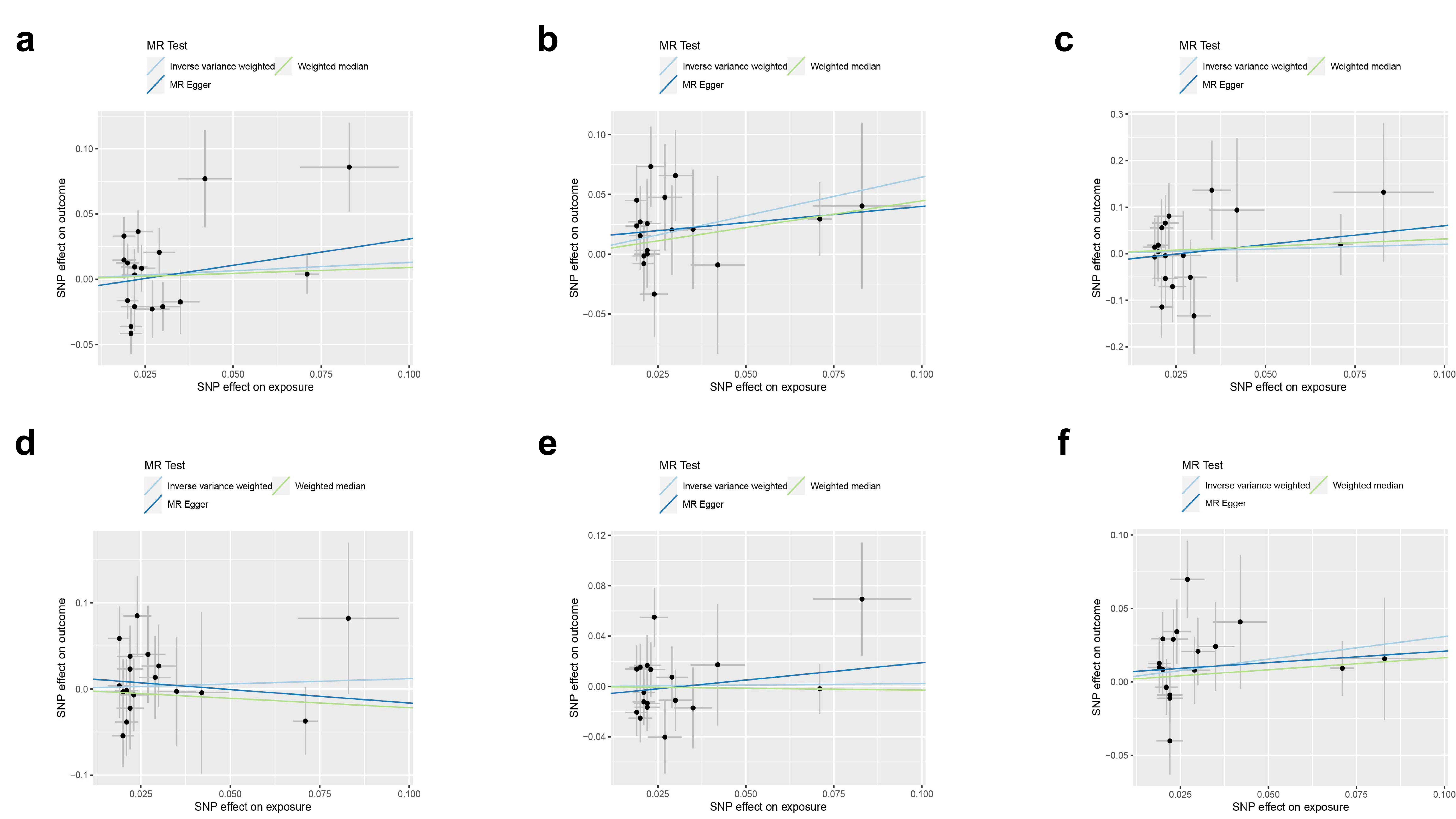

Supplement: Supplementary file 1 — Figures S1–S10 Tables S1–S10 Data S1 [file JCMM-29-e70329-s001.zip › jcmm70329-sup-0001-FigureS1.jpg]

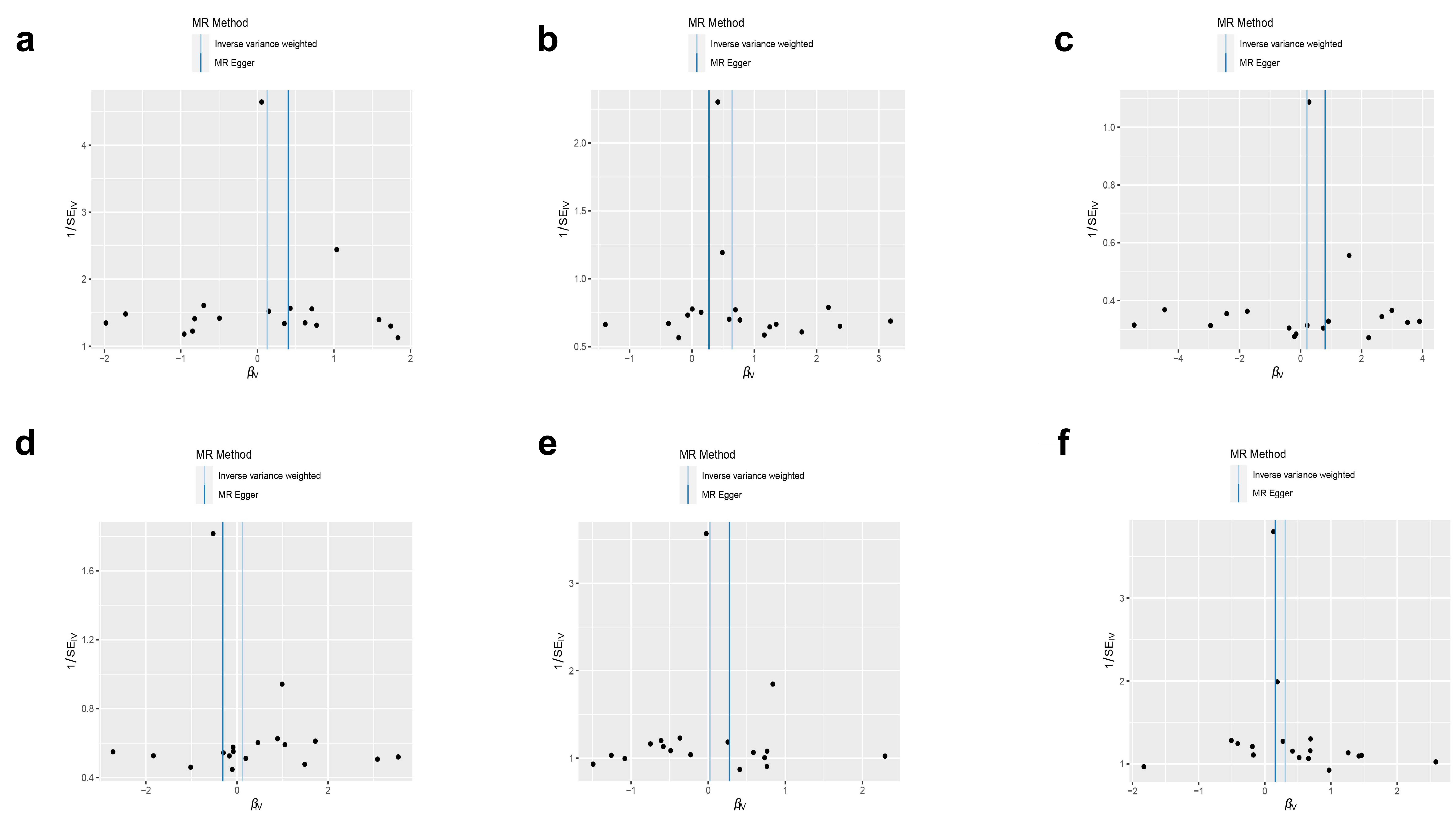

Supplement: Supplementary file 1 — Figures S1–S10 Tables S1–S10 Data S1 [file JCMM-29-e70329-s001.zip › jcmm70329-sup-0002-FigureS2.jpg]

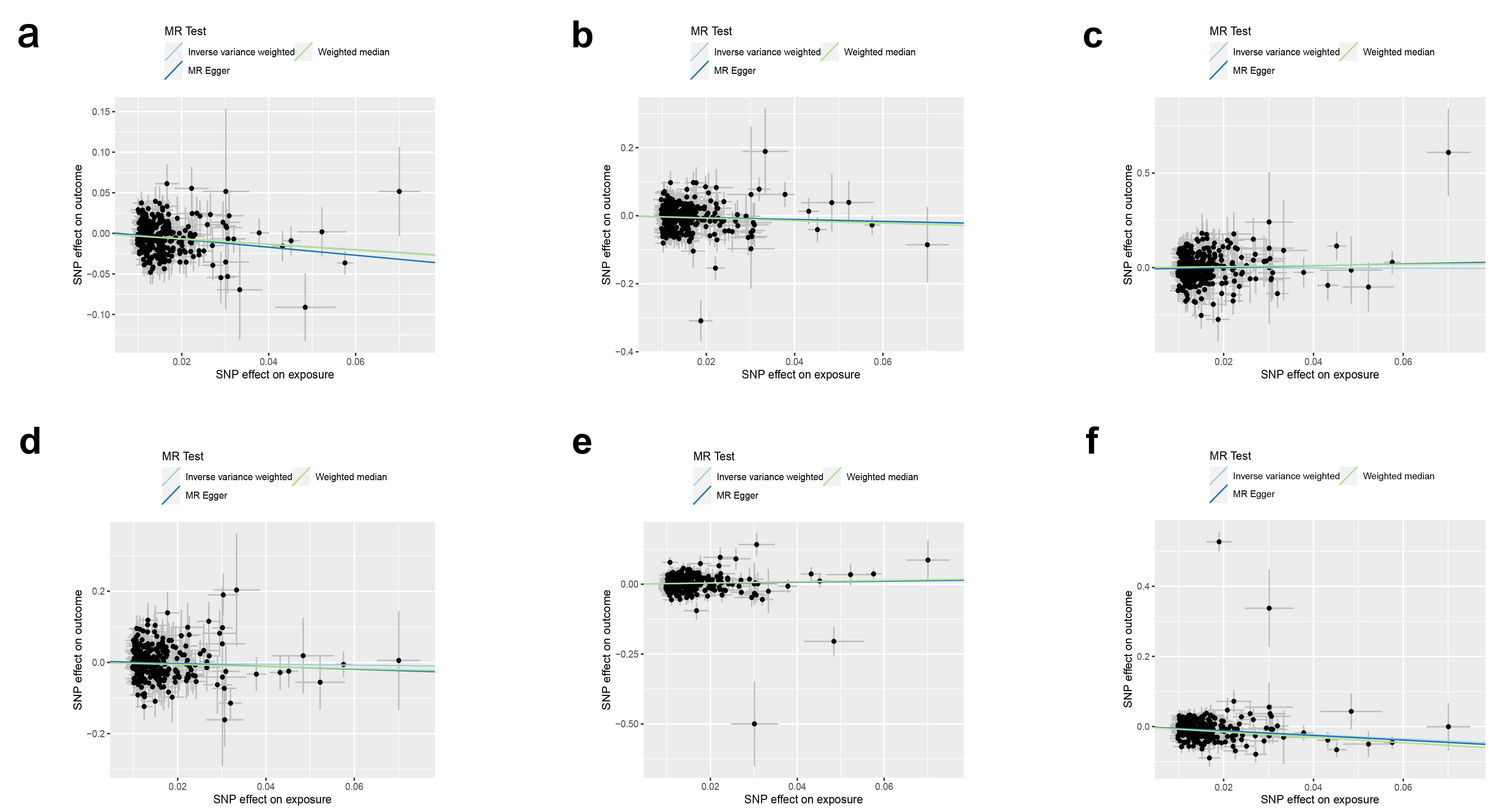

Supplement: Supplementary file 1 — Figures S1–S10 Tables S1–S10 Data S1 [file JCMM-29-e70329-s001.zip › jcmm70329-sup-0003-FigureS3.jpg]

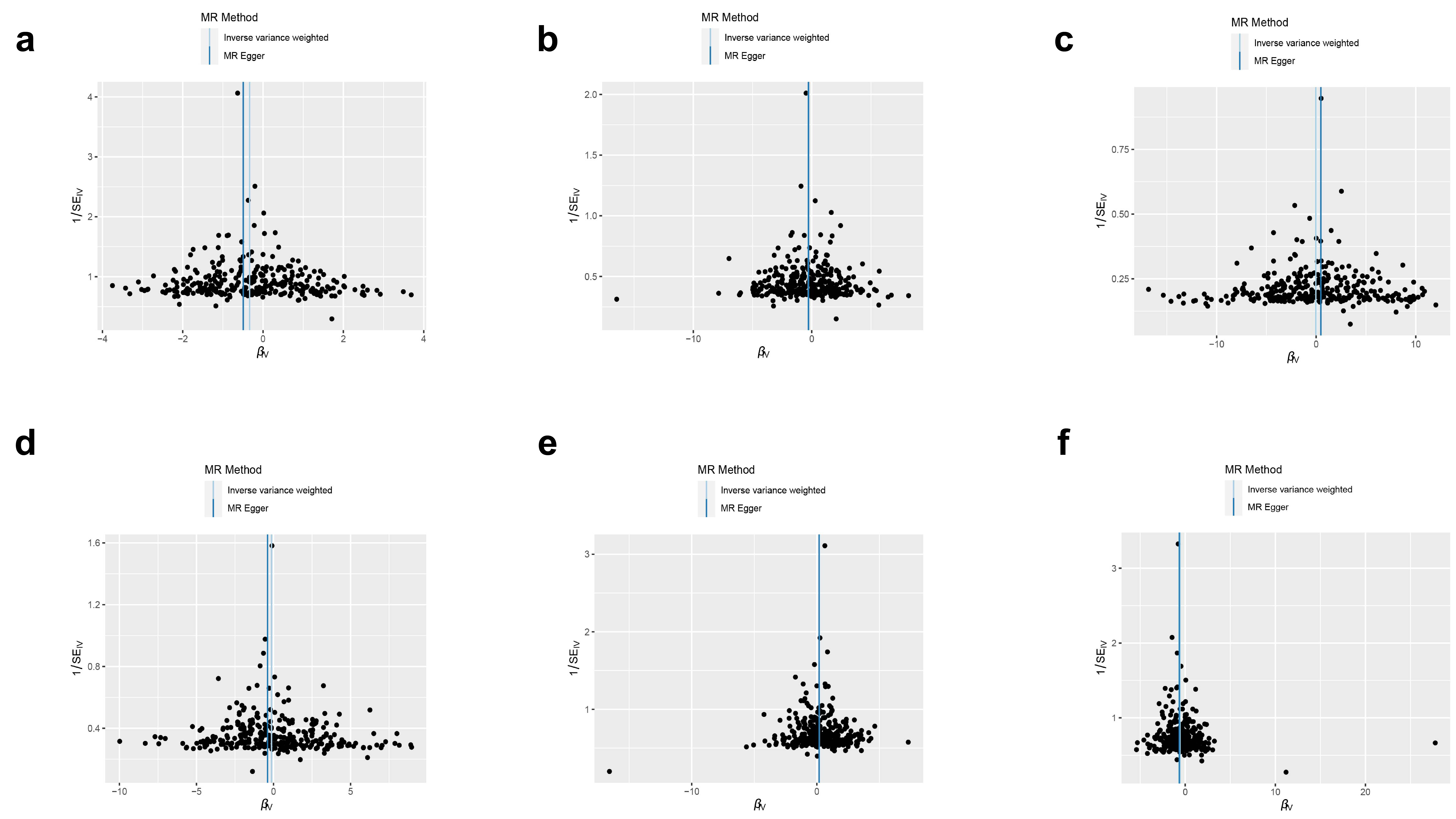

Supplement: Supplementary file 1 — Figures S1–S10 Tables S1–S10 Data S1 [file JCMM-29-e70329-s001.zip › jcmm70329-sup-0004-FigureS4.jpg]

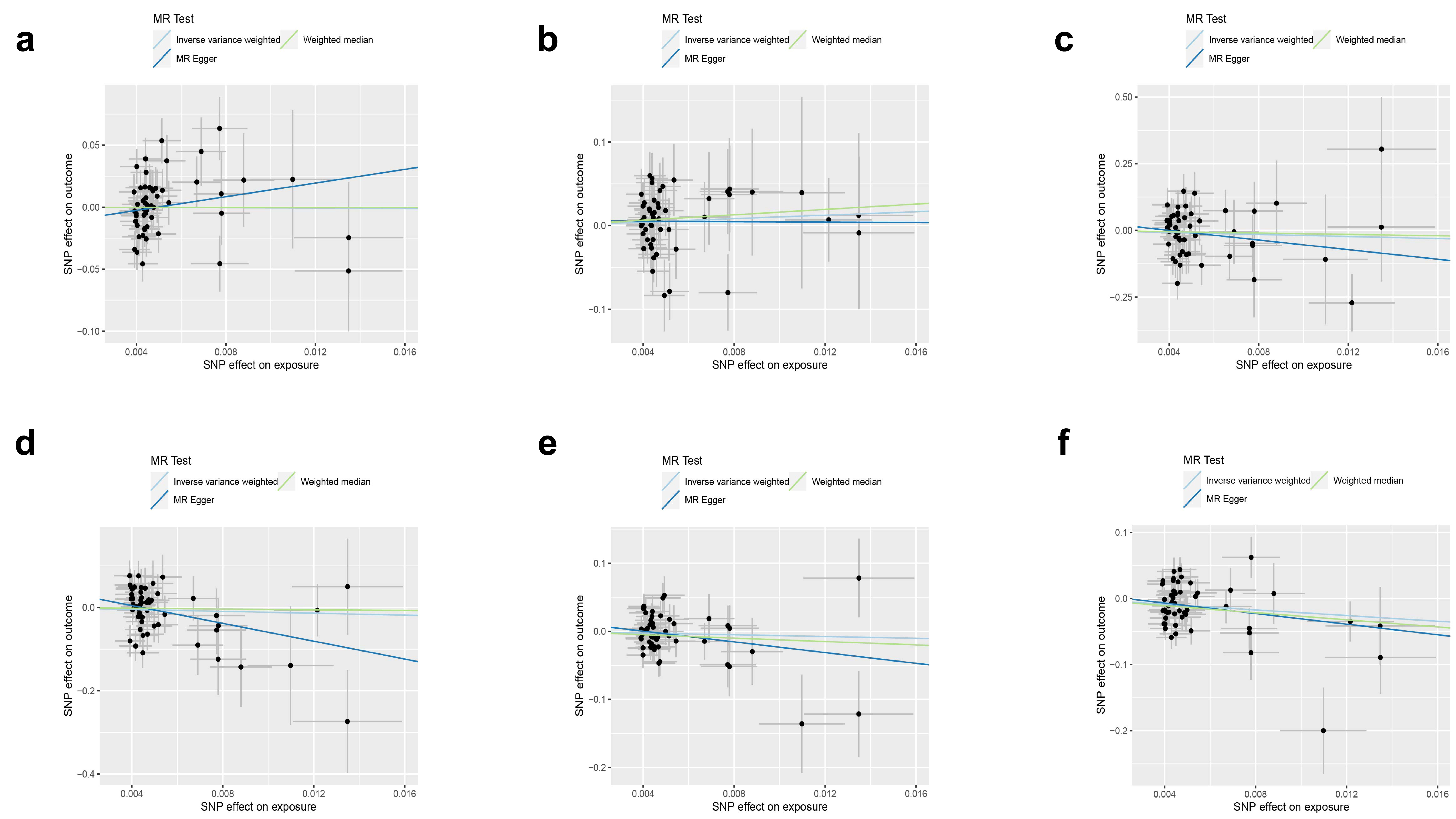

Supplement: Supplementary file 1 — Figures S1–S10 Tables S1–S10 Data S1 [file JCMM-29-e70329-s001.zip › jcmm70329-sup-0005-FigureS5.jpg]

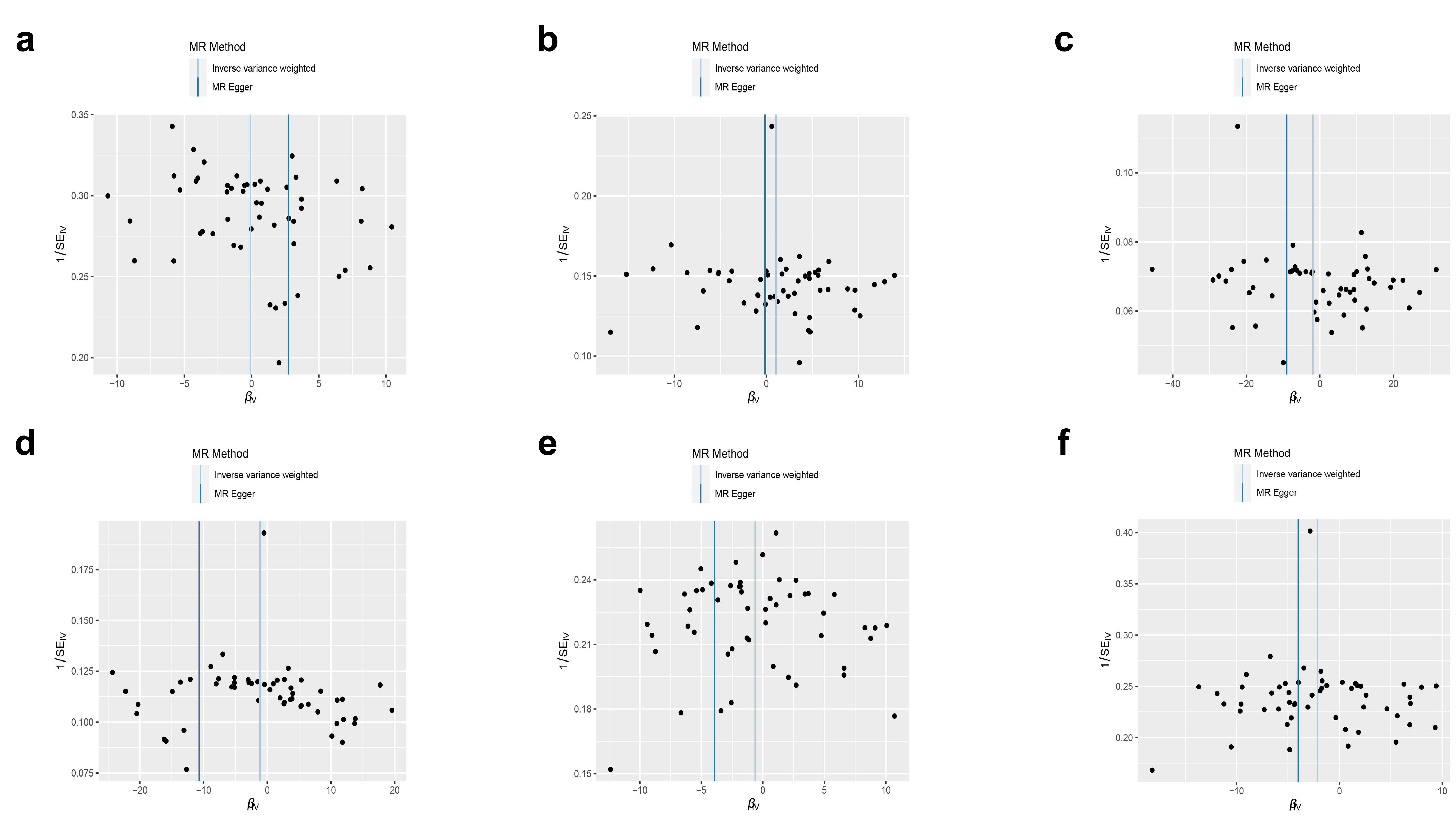

Supplement: Supplementary file 1 — Figures S1–S10 Tables S1–S10 Data S1 [file JCMM-29-e70329-s001.zip › jcmm70329-sup-0006-FigureS6.jpg]

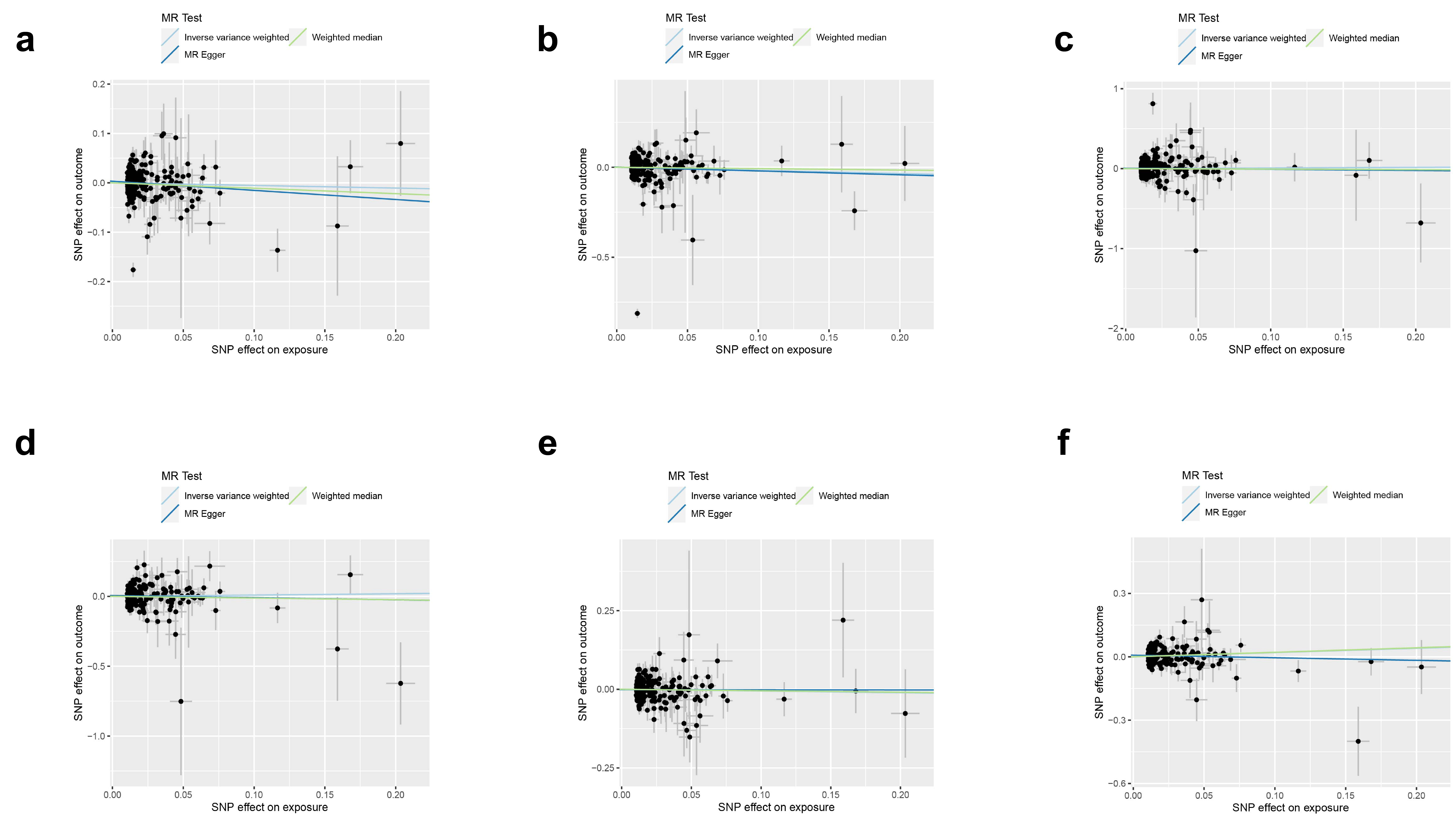

Supplement: Supplementary file 1 — Figures S1–S10 Tables S1–S10 Data S1 [file JCMM-29-e70329-s001.zip › jcmm70329-sup-0007-FigureS7.jpg]

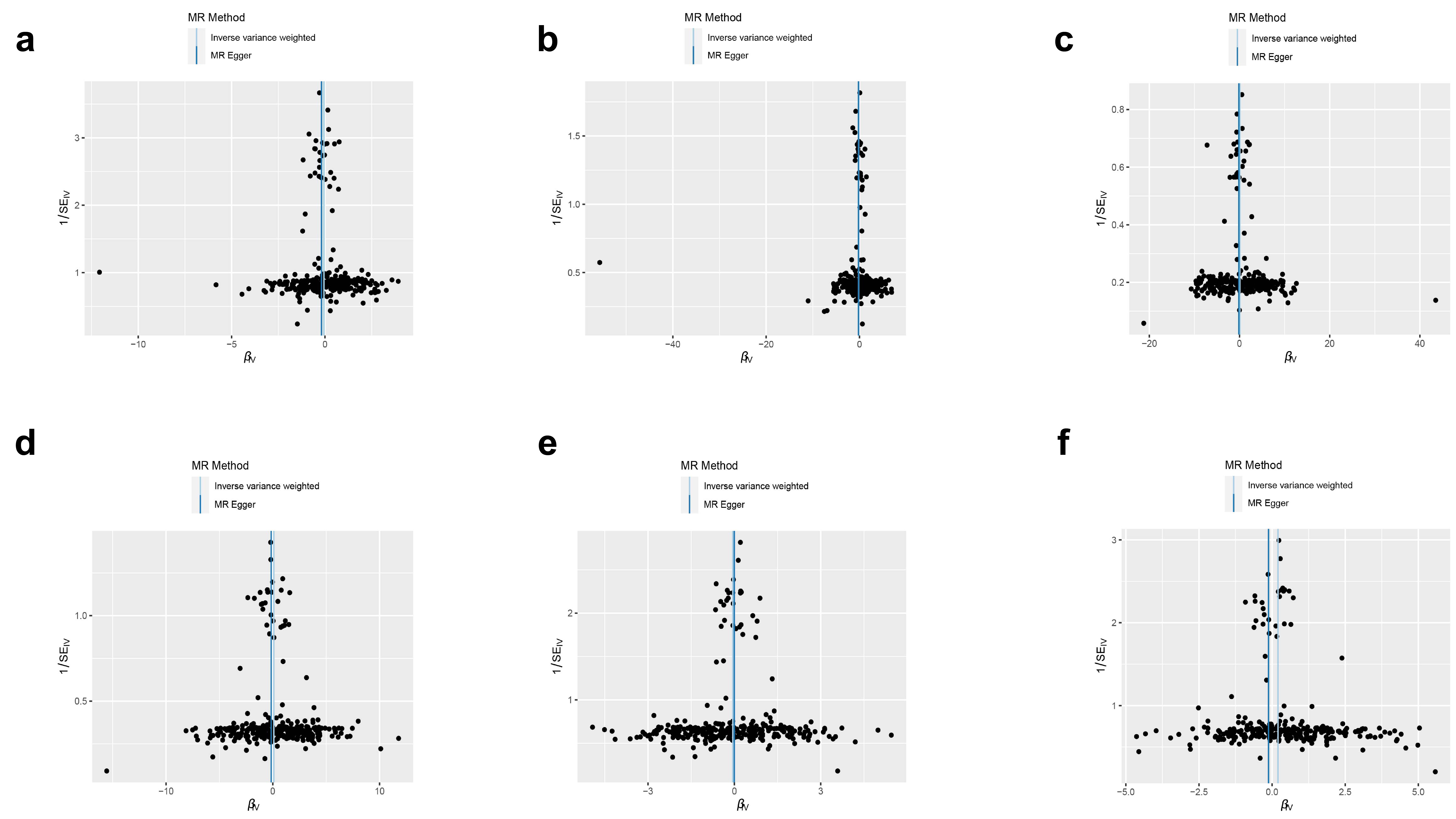

Supplement: Supplementary file 1 — Figures S1–S10 Tables S1–S10 Data S1 [file JCMM-29-e70329-s001.zip › jcmm70329-sup-0008-FigureS8.jpg]

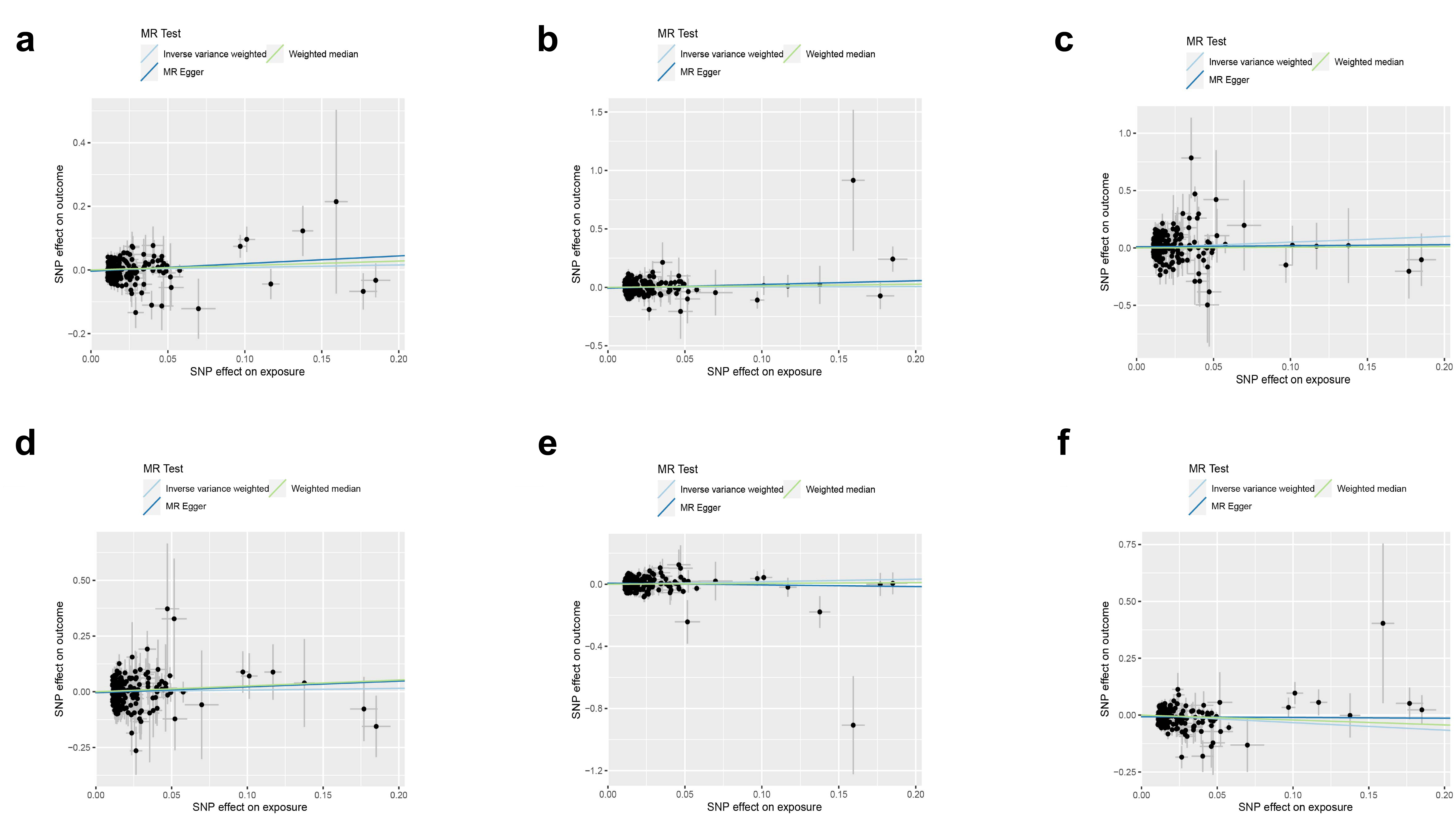

Supplement: Supplementary file 1 — Figures S1–S10 Tables S1–S10 Data S1 [file JCMM-29-e70329-s001.zip › jcmm70329-sup-0009-FigureS9.jpg]

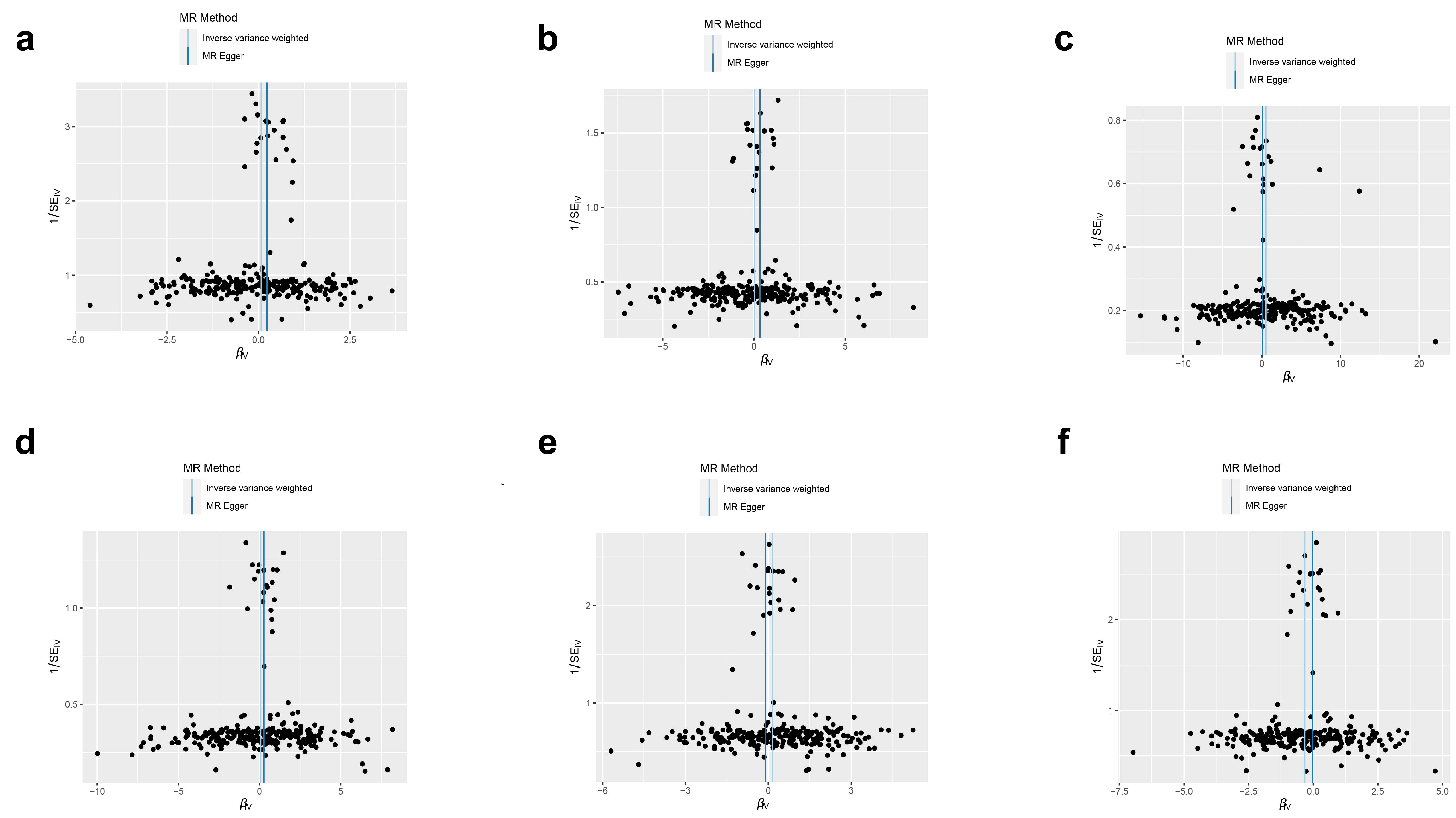

Supplement: Supplementary file 1 — Figures S1–S10 Tables S1–S10 Data S1 [file JCMM-29-e70329-s001.zip › jcmm70329-sup-0010-FigureS10.jpg]
